# Efficacy of Do-It-Yourself air filtration units in reducing exposure to simulated respiratory aerosols

**DOI:** 10.1016/j.buildenv.2022.109920

**Published:** 2022-12-18

**Authors:** Raymond C. Derk, Jayme P. Coyle, William G. Lindsley, Francoise M. Blachere, Angela R. Lemons, Samantha K. Service, Stephen B. Martin, Kenneth R. Mead, Steven A. Fotta, Jeffrey S. Reynolds, Walter G. McKinney, Erik W. Sinsel, Donald H. Beezhold, John D. Noti

**Affiliations:** a Health Effects Laboratory Division, National Institute for Occupational Safety and Health, Centers for Disease Control and Prevention, 1000 Fredrick Lane, Morgantown, WV, 26508, USA; b Respiratory Health Division, National Institute for Occupational Safety and Health, Centers for Disease Control and Prevention, 1000 Fredrick Lane, Morgantown, WV, 26505, USA; c Division of Field Studies and Engineering, National Institute for Occupational Safety and Health, Centers for Disease Control and Prevention, Cincinnati, OH, 45226, USA

**Keywords:** Do-it-yourself air cleaner, Indoor air quality, Inhalation exposure, Ventilation, Air filters, Aerosols

## Abstract

Many respiratory diseases, including COVID-19, can be spread by aerosols expelled by infected people when they cough, talk, sing, or exhale. Exposure to these aerosols indoors can be reduced by portable air filtration units (air cleaners). Homemade or Do-It-Yourself (DIY) air filtration units are a popular alternative to commercially produced devices, but performance data is limited. Our study used a speaker-audience model to examine the efficacy of two popular types of DIY air filtration units, the Corsi-Rosenthal cube and a modified Ford air filtration unit, in reducing exposure to simulated respiratory aerosols within a mock classroom. Experiments were conducted using four breathing simulators at different locations in the room, one acting as the respiratory aerosol source and three as recipients. Optical particle spectrometers monitored simulated respiratory aerosol particles (0.3–3 μm) as they dispersed throughout the room. Using two DIY cubes (in the front and back of the room) increased the air change rate as much as 12.4 over room ventilation, depending on filter thickness and fan airflow. Using multiple linear regression, each unit increase of air change reduced exposure by 10%. Increasing the number of filters, filter thickness, and fan airflow significantly enhanced the air change rate, which resulted in exposure reductions of up to 73%. Our results show DIY air filtration units can be an effective means of reducing aerosol exposure. However, they also show performance of DIY units can vary considerably depending upon their design, construction, and positioning, and users should be mindful of these limitations.

## 1. Introduction

SARS-CoV-2, the virus that causes COVID-19, spreads in the human population by respiratory aerosols and droplets released during exhalatory events, such as breathing, coughing, talking, singing, and sneezing [1–3]. Aerosols and droplets emitted by humans can range in size from tens of nanometers to a millimeter or more (aerosols are defined as airborne particles <100 μm in diameter while droplets are >100 μm) [4–6]. Smaller aerosols (~<10 μm) can disperse quickly and remain suspended in indoor environments for minutes up to several hours [7,8]. The EPA has estimated the average American spends 90% of their time indoors [9] and when the time indoors is associated with groups or events, the probability of viral transmission increases [10]. The potential exposure risk is intensified with increases in the duration of the activity and number of individuals involved and can be compounded by inadequate ventilation [11]. When infectious sources are known or suspected to be present, additional precautions can be adopted to mitigate the risk. However, infected individuals who are pre-symptomatic or asymptomatic also can shed significant amounts of SARS-CoV-2 virus and thus expose others to infectious materials even when attempts are made to screen for symptoms. One study found that 41% of infected individuals were asymptomatic at the time of testing [12]. Research has shown that asymptomatic individuals can spread SARS-CoV-2 [13], making it difficult to slow or stop the spread during indoor events without establishing mitigation strategies.

The U.S. Centers for Disease Control and Prevention (CDC) recommends a combination of risk-based strategies to aid in reducing transmission of SARS-CoV-2 in indoor environments that include vaccinations, universal mask wearing, and improved room ventilation [10]. COVID-19 vaccines can help reduce hospitalizations and death, however long-term immunity is still not fully known and there are risks of breakthrough infections with waning immunity to the vaccine and with virus mutations [14,15]. Laboratory testing has shown that masks, if worn correctly, are 60–95% effective at reducing exposure through source control, but the efficacy of masks can be limited by the type of mask, improper mask fit and non-compliance [16–18]. Physical distancing has been shown to be important when individuals are facing each other less than 1.8 m apart indoors [19,20]. Beyond physical distance, ventilation in a room becomes the dominant contributor to aerosol dispersion and, therefore, personal exposure [21,22]. Increasing indoor ventilation by increasing outdoor and total airflow through heating, ventilation, and air conditioning (HVAC) systems, using more efficient central HVAC filters [23], opening windows to increase natural ventilation [24], and/or adding supplemental air filtration systems help reduce exposure [25]. Older buildings are not always capable of increasing HVAC flow without renovations that are costly and time consuming [26]. Opening windows (natural ventilation) is an easy solution, but with drawbacks such as increasing heating and cooling costs and pulling in dust, pollen, and other contaminants that may result in other harmful respiratory effects [27,28]. Natural ventilation can be highly variable and is dependent on several factors including but not limited to number of open windows, location of windows in the room to create cross ventilation, and outside conditions [24]. Some structures have sealed windows, thus precluding this as an option for increasing ventilation.

A United States Government Accountability Office Report to Congressional Addressees concluded that an estimated 41% of school districts in the United States, representing approximately 36,000 schools nationwide, require updates or replacement of their HVAC systems [26]. Since updating or replacing existing HVAC systems can be fiscally untenable or require long-term renovation, the CDC and ASHRAE have recommended the use of portable high efficiency particulate air (HEPA) cleaner units to supplement the HVAC systems for clearance of potentially infectious aerosols [29,30]. The HEPA filters in these units are designed to remove at least 99.97% of all airborne particles that pass through the unit. Thus, portable HEPA air cleaners can provide a rapidly deployable alternative to increasing effective ventilation rates where renovating existing HVAC systems is not immediately practical. In three studies using mechanical breathing simulators, the use of commercially available portable HEPA air cleaners that were sized to provide 5 air changes per hour (ACH) of filtered air led to a >50% reduction in exposure to simulated exhaled aerosols [21,31,32]. Another study using a calculation model found that the addition of portable HEPA filtration units sized to provide 5.4 ACH can help reduce transmission of SARS-CoV-2 [33]. However, the use of HEPA filtration units also has notable disadvantages, including high initial purchase costs, high costs of filter replacement and noise levels that can surpass 50 dB [34]. The American National Standards Institute (ANSI) recommends classroom background noise should not exceed 50 dB with acceptable levels between 35 and 45 dB [35].

Due to supply shortages and the high costs of HEPA air cleaners, homemade or Do-It-Yourself (DIY) air filtration units became popular during historically large wildfire outbreaks in the western United States due to their efficacy in reducing smoke and other airborne particles [36]. These units were often constructed using readily available house fans and residential HVAC filters. Several variations of the DIY units were used with a single filter, either secured to the front or back of the fan. The units were inexpensive and straightforward to create from supplies available at local hardware or department stores. However, testing the filtration efficiency of the DIY units was rare. Filters for home HVAC systems also vary greatly in efficiency ratings. DIY air filtration units have also been employed by the public to reduce exposure to infectious aerosols due to their low cost, ease of construction and ready availability compared with the higher cost and limited availability of commercial HEPA air cleaners [37]. However, comprehensive evaluations of these DIY units in reducing respiratory aerosols exposure in a real-world scenario have been limited to date.

The Ford Motor Company and Lasko Products, LLC developed and produced a simple DIY air filtration kit and shipped 20,000 units to organizations in underserved communities, including school districts in Los Angeles, New York City and throughout Southeast Michigan to help slow the spread of COVID-19 [38]. The kits consisted of a cardboard box that contained a 51 cm box fan, a 51 × 51 × 10 cm minimum efficiency reporting value (MERV) 13 filter and a set of instructions to convert the box into a holder for the fan and filter (Fig. 2A) [39]. Research using a computational fluid dynamics (CFD) model demonstrated that, with a room volume of 150 m^3^ and a flowrate of 0.2 m^3^/s through each air filtration unit, one Ford DIY air filtration unit could extract 14–75% of airborne particles and two units could extract 53–84% in 50 min depending on the location of the air filtration units relative to an aerosol source [40].

Dr. Richard Corsi (Dean of Engineering at the University of California, Davis) and Jim Rosenthal (CEO of Tex-Air Filter) were inspired to create an air filtration unit made with five filters taped together to form a cube with a box fan taped to the top. The use of multiple filters reduced the airflow resistance and greatly increased the amount of air flowing through the filtration unit [41]. This air filtration device with five filters was dubbed the “Corsi-Rosenthal Box” or CR Box, and a version using four filters was dubbed the “Comparetto Cube” (Fig. 2B) [41]. These “DIY air filtration cubes” are simple to construct and less expensive than commercial HEPA air cleaners. Through a grassroots movement by social media and news outlets, thousands of DIY air filtration cubes have been built for use in schools and other public building [41]. A study of the DIY air filtration cubes examined different configurations of DIY air filtration units and compared their estimated clean air delivery rate (CADR) to portable HEPA air cleaners. The authors concluded that the DIY air filtration cube performed similarly to commercial portable HEPA air cleaners based on their estimated CADR values [42,43].

Our investigation examined the efficacy of DIY air filtration units in reducing recipient exposure to simulated respiratory aerosols within a mock classroom. We used a custom-built respiratory aerosol simulator that breathes and exhales aerosol particles and three breathing simulators to simulate the combined effects of the room ventilation system, DIY air filtration units, and human respiratory activities on exposure to respiratory aerosols. Seven box style fans were purchased based on commercial availability and evaluated using five performance parameters to determine which parameters were most important in reducing exposure when incorporated in a DIY unit. Two fans with the highest and lowest airflow rates were subsequently evaluated in two DIY unit configurations using either 2.5 or 5 cm deep MERV 13 filters. DIY air filtration units were tested with the central HVAC system set at 2 air changes/hour (ACH) to represent a classroom with low ventilation. Results of the investigation provide a better understanding of DIY units and their potential to reduce exposure to infectious aerosols that can transmit SARS-CoV-2 and other diseases.

## 2. Materials and methods

### 2.1. Mock classroom layout and room ventilation

**Fig. 1.**
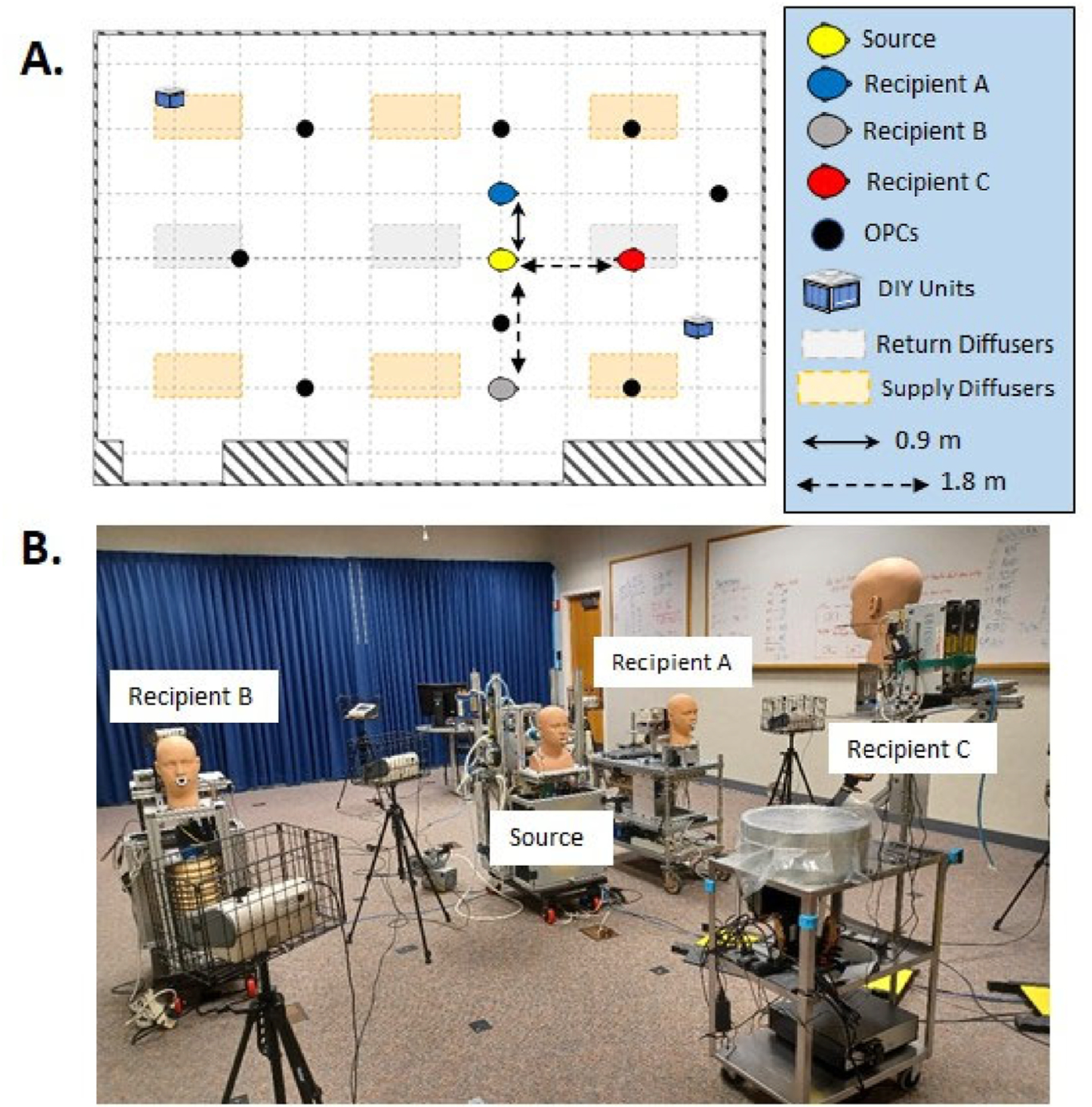
Mock classroom. (A) Layout of the room with positions of breathing simulators, optical particle counters (OPCs) and DIY air filtration units. Gridlines are evenly spaced at 0.9 m. Black dots denote locations of the TSI 3330 OPCs, while the Grimm OPCs were co-located with the recipient headforms. The orange rectangles denote the location of the HVAC system supply slot diffusers. Gray rectangles denote the location of the HVAC system return air diffusers. (B) Photo of the room set up with the locations of Recipient A, B and C breathing simulators and the Source respiratory aerosol simulator. Curtains at the back of the room covered fixed interior windows; the curtains were opened and drawn to the side of the room during experiments. (For interpretation of the references to color in this figure legend, the reader is referred to the Web version of this article.)

A conference room was used as the mock classroom for these experiments. It measured 6.6 m wide by 9.1 m long with a height from floor to ceiling of 3 m for an air volume of 180 m^3^. The airflow to the room originated from the building’s Air Handling Unit (AHU) which mixes return air with fresh outside air. The AHU that supplies air to the room is a large system serving multiple areas. The total supply air passed through a set of prefilters (HC MERV 10 Pleated Air Filter; Filtration Group; Mesa, AZ) and a MERV 13 V-Bank filter (DuraMAX 4v; Koch Filter Corporation; Louisville, KY) before being supplied to the room through six 0.6 m × 1.2 m slot diffusers, all controlled by the same variable-air-volume (VAV) box. The slot diffusers were evenly distributed with three diffusers along each longitudinal wall. The return air entered the ceiling plenum through three 0.6 m × 1.2 m diffusers located through the midline of the room (Fig. 1A and B). All furniture was removed from the room to avoid the possibility of dead air spaces.

### 2.2. HVAC clearance rates

Indoor ventilation and filtration rates are commonly expressed in terms of air changes per hour (ACH), defined as the room volume divided by the airflow. Ventilation and filtration rates are often determined by measuring the clearance rate, which is the rate at which a tracer is removed from the room air. In our study, the HVAC system clearance rates were determined using three methods: an HVAC measurement/calculation method based on the measured total HVAC clean air supply rate (because the room was under positive pressure, the supply air was measured instead of the return air), a tracer gas decay method using sulfur hexafluoride tracer gas, and an aerosol decay method using potassium chloride (KCl) aerosols. Full details of the methods are described in a prior study conducted in this conference room [21]. All experiments were conducted with the room HVAC system and VAV box set to provide a constant 2 ACH.

### 2.3. Breathing simulators and masking

**Fig. 2.**
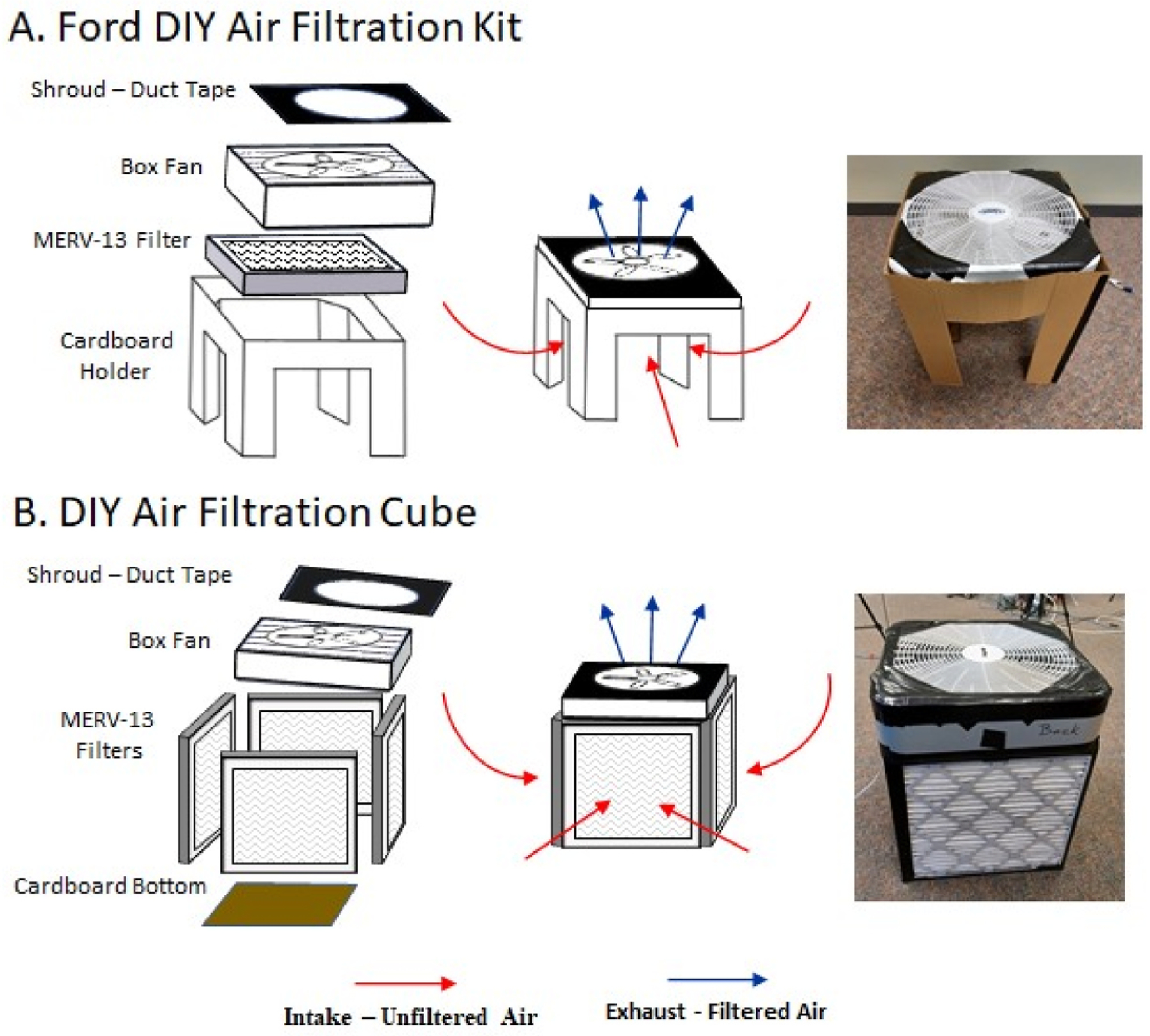
Do-it-Yourself air filtration units. (A) The modified Ford DIY air filtration Unit (DIY Ford) constructed with one filter placed inside the cardboard holder with a shrouded fan on top. (B) DIY air filtration cube (DIY Cube) constructed with four filters forming a cube, taped together with duct tape and the fan taped to the top of the filter cube. In both configurations the filter airflow directional arrows were pointed towards the fan, so that air traveled through the filter first and then through the fan.

To examine the efficacy of the DIY air filtration units in reducing exposure to potentially infectious respiratory aerosols, one aerosol-producing Source breathing simulator and three Recipient breathing simulators were positioned to simulate a mock classroom (or lecture) setting (Fig. 2). The Source and Recipient simulators are not heated and breathe room temperature air as described previously [16]. The respiratory aerosol source simulator simulated a person who was exhaling aerosol particles into the room. The test aerosol was produced using a solution of 14% potassium chloride (KCl) in a single-jet Collison nebulizer (BGI; Butler, NJ) at 103 kPa (15 lbs./in^2^). The aerosol passed through a diffusion drier (Model 3062; TSI; Shoreview, MN), mixed with dry filtered air and was neutralized using a bipolar ionizer (Model HPX-1; Electrostatics; Hatfield, PA). The aerosol mixing occurred in an elastomeric bellows which served as the mixing chamber and simulated lung of the Source simulator. During the experiments, the nebulizer was continuously cycled 20 s on and 40 s off to prevent the aerosol concentration in the room from exceeding the upper concentration limit of the aerosol particle counters.

Each of the three Recipient simulators was equipped with an optical particle counter (OPC; Model 1.108; Grimm Technologies, Inc.; Douglasville, GA, USA) connected to a stainless-steel sampling tube extending from the back of the head to an opening directly adjacent to the mouth, which measured the exposure to the simulated respiratory aerosols. The OPCs had a size measurement range of 0.3–3.0 μm with a sampling frequency of 1 Hz. The sampling tube was located such that, when a mask was worn by the simulator, the sampling tube was under the mask. One Recipient simulator representing a teacher or speaker (called Recipient C) was positioned in a standing height near the front of the room with the mouth position and OPC sampling tube 152 cm from the floor. Recipient C was positioned near the midline of the room and between the intake slot vents in the ceiling. The Source was positioned within the foremost row of the audience/participant area in a sitting position and 1.8 m directly in front of Recipient C with the center of the month 101 cm above the floor. Recipients A and B were adjacent to the Source simulator, with A positioned 0.9 m to the left of and B positioned 1.8 m to the right and with the mouth and OPC sampling tube positioned 101 cm from the floor.

All Source and Recipient simulator head forms were covered with a synthetic elastomer to simulate the pliability and texture of human skin and did not include nostril openings. The Source head form was from Hanson Robotics (Plano, Texas, USA), while Recipient head forms were from Crawley Creatures Ltd (Model: Respirator Testing Head Form 1; Buckingham, UK). The Source and Recipient A and B simulators breathed using a computer-controlled linear motor affixed to elastomeric bellows to simulate lungs. The breathing cadence was calibrated to a tidal volume of 1.25 L/breath and 12 breaths/minute with a minute ventilation rate of 15 L/min corresponding to the ISO standard for females performing light work [44]. Recipient C breathed using a commercial respiratory simulator (Warwick Technologies Ltd.; Warwick, UK) [16] with a sinusoidal breathing waveform calibrated to 21.5 breaths/minute with a minute ventilation rate of 26 L/min, which is approximately the average of the ISO standards for males and females engaged in moderate work [44].

Face masks were 3-ply cotton cloth masks with ear loops (Defender; HanesBrands Inc.; Winston-Salem, NC, USA). Experiments were conducted with all simulators either unmasked or masked (universal masking). Mask fit was determined using the PortaCount Pro+ (Model 8038; TSI Corporation; Shoreview, MN) in N99 mode as per manufacturer’s instructions and the results are in the Supplemental material.

### 2.4. Box fan specifications

The fans used in the study were purchased based on two considerations. First, the fans were listed as 20′′ box fans, which is the style of fans used by the public to construct DIY units. Second, the selected fans were easily obtainable at the time of the study. No attempt was made to survey all possible models of fans or to select fans based on a particular performance criterion. Fan power and/or airflow criteria could not be used to select fans since very few fans are tested against a performance standard and most fan manufactures do not report specifications in their product literature. A convenience sample of seven new 51 cm (20”) box style fans were purchased for evaluation (listed alphabetically): Air King model 4CH71G/9723G (Fan A), Comfort Zone model CZ200A (Fan B), Genesis model G20BOX-WHT (Fan C), Hurricane model HGC736501 (Fan D), Lasko model B20200 (Fan E), Lasko Premium model 3723 (Fan F), and Pelonis model FB50–16H (Fan G). All fans were listed as residential fans except Fan A which was labeled as a commercial grade fan. A shroud made of duct tape (Fig. 2A and B) was attached to the corners of the fan chassis that extended beyond the end of the fan blades on the outflow side of the fan to increase airflow efficiency [45]. The following performance parameters were evaluated for each fan while running at high and low speeds: airflow in cubic feet per minute (CFM), noise in decibels (dB), fan current (ampere), fan power (Watts) and fan blade revolutions per minute (RPM). Parameters were measured on the shrouded fan alone and two configurations of DIY air filtration units with 2.5 and 5 cm filters. Descriptions of the two DIY air filtration unit configurations are illustrated in Fig. 2 and described in section 2.5 below. Fans were allowed to operate for at least 1 min prior to acquiring all measurements to achieve full operational speed.

Airflow for each fan was determined using an Alnor^®^ LoFlo Balometer^®^ with a 0.6 m × 0.6 m Capture Hood (Model EBT731; TSI Corporation). A 70 × 70 cm piece of double strength cardboard with a 50 cm circular hole in the middle was centered on top of the fan. The balometer was then placed on top of cardboard and the airflow was measured.

A real-time octave band analyzer (Extech model 407790; Extech/FLIR Systems; Nashua, NH) was used to measure the decibel A scale (dBA) levels of the fans and DIY units placed in the same location at the front of the room. The room HVAC system was set at a constant 2 ACH for all noise measurements and all doors were closed to the room. To minimize location effects of the DIY units on noise, equivalent continuous sound pressure level (L_eq_) measurements for 5 s were acquired at the eight OPC locations and then averaged together for a room mean noise level. This procedure was used to obtain a background noise level with the HVAC system set at 2 ACH and no DIY units operating. Additionally, noise levels were measured for DIY air filtration cubes operating simultaneously in the front and back of the room constructed with Fan A and B with both 2.5 and 5 cm filters.

Electrical current measurements were obtained by plugging the fan’s electrical cord into a line splitter and measuring current using a digital Volt multimeter (Fluke Model 189; Fluke Corporation; Everett, WA) with an AC current clamp (Fluke Model i800). Prior to turning the fan on to take measurements, the current clamp was placed on the line splitter to obtain a background current reading, which was subtracted for the operating reading. The voltage of the electrical outlet was measured with the multimeter prior to taking current measurements. Fan power in Watts was determined by the Watts Law Formula; P=V*C (P is power in Watts, V is voltage in Volts and C is current in Amperes). The fan blade rotation rate was measured with a non-contact optical tachometer (Monarch model PT99; Monarch Instrument; Amherst, NH).

### 2.5. Description of DIY air filtration units

DIY air filtration units were assembled using 51 × 51 cm (20′′ × 20”) MERV 13 Air Handler pleated filters (W.W. Grainger, Inc.; Lake Forest, IL) with pleats that were either 2.5 or 5 cm thick (Supplemental Table S1 – Filter Specifications). The MERV rating is a performance rating for HVAC filters based on ANSI/ASHRAE Standard 52.2–2017, with higher numbers indicating higher filtration efficiencies. A MERV 13 rating means that the filter removes ≥50% of aerosol particles with a diameter of 0.3–1 μm, ≥85% of 1–3 μm particles, and ≥90% of 3–10 μm particles. MERV 13 filters were selected because, early in the COVID-19 pandemic, ASHRAE recommended that HVAC filters in non-healthcare facilities be upgraded to MERV 13 filters if possible, or otherwise to the highest rated filter an HVAC system can accommodate [46].

Fans A and B were each used to construct two configurations of DIY units, yielding four different DIY unit configurations. The first two unit configurations were a modified version of the Ford/Lasko DIY air filtration unit (Fig. 2A) with either a 2.5 cm filter (configuration 1) or 5 cm filter (configuration 2) [39]. The design was modified from the original by increasing the length of the feet of the cardboard holder to give a total height of 61 cm and orienting the filter and fan, so the direction of airflow was upwards to place the fan at the same height and airflow direction as the DIY air filtration cubes. The original Ford kits were supplied with 10 cm thick filters, but this study used 2.5 and 5 cm filters because they are more widely available. The third and fourth configurations were DIY air filtration cubes (Fig. 2B) built with four filters that were either 2.5 cm thick (configuration 3) or 5 cm thick (configuration 4). Four filters were used so that the units could be placed directly on the floor on a cardboard base. The filters were orientated with the filter directional airflow arrows pointed inward so that the direction of airflow was inward through the filters. Units were taped together along the entire edge of the filters with duct tape (Gorilla Heavy Duty Black, 603560) and a 51 cm × 51 cm cardboard base was taped to the bottom of the filters. A box fan was taped to the top of the cube along all edges with the direction of airflow upwards. All gaps between filters/fan and any holes in the fan chassis were sealed with duct tape to ensure air was drawn through the filters and not bypassing the filters through leaks.

### 2.6. Aerosol clearance rates using the DIY air filtration units

The aerosol concentration decay method was used to determine the effective ACH for each DIY air filtration unit. Using a 3-jet Collison nebulizer, the meeting room was dosed with aerosols from a 14% KCl solution in distilled water for 20 min. A 64 cm diameter pedestal-base vane axial fan provided mixing prior to aerosol measurement. Aerosol concentrations were quantitated for a minimum of 20 min during the aerosol decay phase. To measure aerosol concentrations, eight OPCs (Model 3330; TSI Corp.) were symmetrically placed in the room at a height of 101 cm corresponding to the sitting height used in this study (Fig. 1A). The OPCs sampled at 1 s intervals and were set to measure aerosol particle number concentrations in three size bins: 0.3–0.4 μm, 0.4–0.5 μm, and 0.5–0.65 μm. The bins were aggregated together for each instrument and fit with an exponential decay curve. and then used to derive the aerosol concentration decay rate to calculate the air exchange rate. Since the air change rate used here is not a traditional air exchange where all air is removed from the room by way of the HVAC system but included both the HVAC system and air filtration through the DIY units, it will be described as an effective air change rate. Effective air change rates were calculated for each of the eight OPCs and then averaged to calculate the mean effective air change rate for each experimental condition.

### 2.7. Test procedure and aerosol measurement

At the start of each test run, residual particles were cleared by increasing the HVAC system ventilation rate to maximum and observing the aerosol concentration over time. When a plateau was reached, the room HVAC system was set to 2 ACH and the DIY air filtration unit(s) turned on for a minimum of 5 min to reach a steady-state airflow pattern. Recipient simulators were also activated to initiate the breathing cycles, while OPC sampling was started to collect the background particle concentrations. At test time zero, the Source simulator was activated to breathe with a sinusoidal waveform of 15 L/min continuously throughout the experiment. Aerosols were generated using a single jet Collison nebulizer filled with 14% w/v KCl in distilled water, with a cadence of 20 s aerosol generation followed by 40 s of no aerosol; the aerosols were produced throughout the 60-min duration of the experiment. Supplemental Figure S1 shows the size distribution graph of the exhaled aerosol. Each experimental condition was performed in quadruplicate.

The room temperature and humidity were monitored in real-time using a temperature and relative humidity probe and data logger (Vaisala Oyj; Vantaa, Finland). Barometric pressure was reported by each TSI OPC. Ambient room conditions data are in the Supplemental material.

### 2.8. Data processing and statistical analysis

Size-binned particle count data and elapsed time reported by each Grimm and Model 3330 OPC were processed using the R Statistical Environment v. 4.0.5 (R Project for Statistical Computing; Vienna, Austria). Bin-specific particle counts for the 180 s preceding the start of aerosol generation were used to estimate the background aerosol concentration, which were then subtracted from OPC particle counts. The mass concentration of aerosol (μg/m^3^) per size bin was calculated by multiplying the bin-specific particle count by the volume of the bin-specific median diameter (assuming the particles were spherical) and then multiplying by 1.984 g/cm^3^ (density of KCl). Note that this conversion from particle counts to particle mass is commonly used but is an approximation. For each OPC, the bin-specific background-corrected aerosol mass concentration was summed across all bins to derive a total aerosol mass concentration per time point. The aerosol mass concentration throughout the experiment was averaged to determine the mean aerosol mass concentration (mean aerosol exposure) which served as the exposure metric for the investigation for each Recipient.

To assess the effect of the DIY units and masking on exposure, a multiple linear regression model was constructed using the log-transformed mean aerosol mass concentration against the binary factor of masking (e.g., no masking and masked with the 3-ply cotton mask) and the effective air change rate measured in decay testing. In order to convert the log-transformed Regression Coefficients to the proportion of remaining exposure per unit increase in the respective variable, the Regression Coefficients and CI95% values were to the power of Euler’s Number (*e*) to back-transform the values to proportion of exposure. A second multiple linear regression model was constructed to assess the effect of the DIY parameters of fan model (Fan A and B), fan speed (low and high), DIY placement (1 unit in back, 1 unit in front, and 2 units), MERV-13 filter width (2.5 cm and 5.0 cm), and DIY design (Ford and cube) on effective air change rate. Point estimates presented in the text, figures, and tables are the arithmetic mean ± 1 standard deviation of the mean aerosol exposure in units of μg/m . Multiple linear regression models and statistical analyses were conducted using the R Statistical Environment. Statistical significance was set at p < 0.05.

### 2.9. Spatial mean mass concentration distribution

Area samples measured from the Model 3330 OPCs were used to generate 2D rasterized overlays of mean mass aerosol concentration. Spatial overlays were performed by inverse distance weight modeling with the “gstat” package in R using the observed data as described in a prior study [21].

## 3. Results

### 3.1. Box fan specifications

Airflows for all fans and DIY air filtration unit combinations as single units are presented in Table 1. The highest airflow measurements for all fans occurred when the fans were shrouded but not attached to the filters since there was no restriction from filters. When all seven fans were incorporated into DIY units, the DIY cubes had the highest airflow rate compared with the Ford DIY units. Additionally, airflow was highest in the DIY units constructed with 5 cm filters. When shrouded only, the fan with the highest airflow was Fan A with 712 CFM on low and 959 CFM on high speed. The lowest airflow occurred with Fan B with 428 CFM at low speed and 625 CFM on high speed. Fans A and B were selected to be incorporated in the DIY units since they had the highest and lowest airflow rates.

The noise levels for each fan averaged over the eight OPC locations are presented in Supplemental Table S2. The background room mean sound level was 36.1 ± 0.5 dBA. The fans with the highest and lowest shrouded airflow measurements on high and low speed (Fans A and B), also had the highest and lowest mean sound levels. The Fan A produced a noise level of 55.8 dBA on low and 62.2 dBA on high speed, while Fan B produced 41.3 dBA on low and 50.5 dBA on high. There were no observable differences in noise levels when the fans were incorporated into any of the DIY configurations compared with fans that were shrouded only. However, when two DIY units were used (one in front and one in the back of the room), noise levels increased by 3–5 dBA compared with running a single unit.

No observable differences between shroud only and DIY configurations were observed for the other fan specifications measured (electric current, power, or blade rotation rate) when incorporated into one of the four DIY configurations. However, it should be noted that fan power and fan blade rotation rate were only weakly correlated with fan airflow rate. For example, the fan with the highest power did not have the highest airflow rate and the fan with the lowest power did not have the lowest airflow rate. Correlation analysis showed only 86% correlation between fan airflow and fan output power and 64% correlation between airflow and blade rotation. These measurements are presented in Tables S3–S5 in the supplemental material.

### 3.2. Clearance rates for the room and DIY air filtration units

The particle decay method was used to determine the effective air change rate of the room with the HVAC system set to a nominal value of 2 ACH. Under this baseline condition, the actual air change rate was 1.89 (SD 0.14) as determined in a prior study [21].

**Fig. 3.**
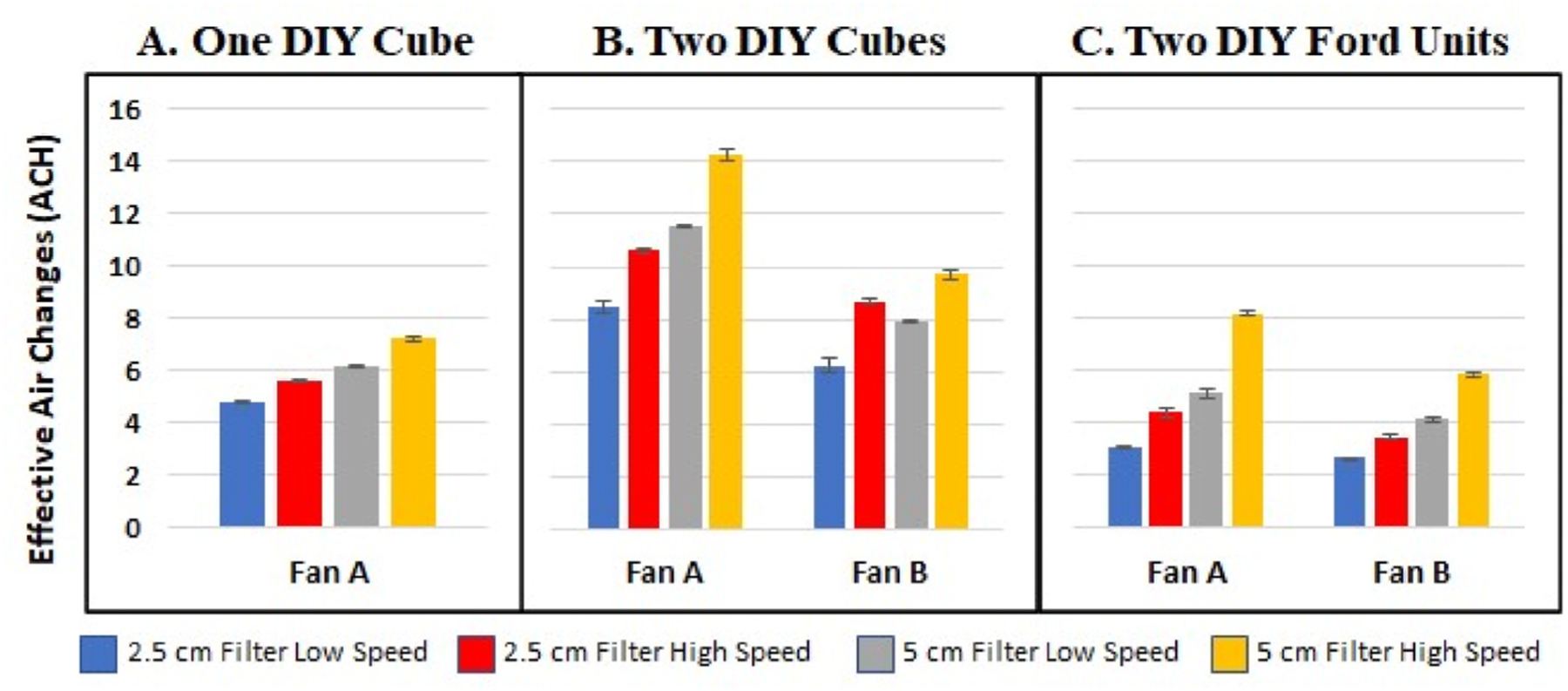
Total air change rate. The effective ACH values shown for the DIY units reflect the combination of the room HVAC system and the filtration effect of the DIY units. For all experiments the room HVAC was operating at a nominal setting of 2 ACH (actual rate 1.89 ACH). (A) Single DIY cube constructed with fan A; (B) Two DIY cubes (constructed with fan A or B), one in the front and one in the back of the room operating simultaneously; and (C) two modified Ford DIY air filtration units (constructed with fan A or B) one in the front and one in the back of the room operating simultaneously. Values represent the mean and standard deviation of three independent measurements.

Fig. 3 shows the total air change rate for the room HVAC system operated in combination with one or two of the DIY units. The following observations are for two units operating simultaneously, one in front and one in the back of the room: When Fan A and B were incorporated into the DIY air filtration units, the air change rates averaged 32% higher with units constructed with Fan A compared with units constructed with Fan B. Comparing filter width, the 5 cm filters averaged 31% higher air change rates than the 2.5 cm filter. The DIY air filtration cube flow rate averaged 121% higher than the modified Ford DIY air filtration unit. DIY air filtration cubes constructed with Fan A and 5 cm filters gave the highest air change rates, providing 4.8 ACH per unit on low speed and 6.2 ACH per unit on high. Combined with the 1.89 ACH from the HVAC system, this resulted in total air change rates of 11.54 ACH on low speed and 14.26 ACH on high. In comparison, Fan B in the same configuration added an additional 3.0 ACH on low and 3.9 ACH on high for each unit.

Using the ACH from each condition, an estimate Clean Air Delivery Rate (CADR) was determined by using the following: CADR = (ACH – 1.89) × 6357/60 where CADR is the Clean Air Delivery Rate in cubic feet per minute (CFM), ACH is air changes per hour determined by the particle decay and including both the HVAC and DIY units, 1.89 is the effective ACH of the HVAC system set at 2 ACH, 6357 is the volume of the room in cubic feet, and 60 is the conversion factor from hours to minutes. The results are presented in Table S6 in the Supplemental Material.

### 3.3. Effects of the DIY units on aerosol exposure

**Fig. 4.**
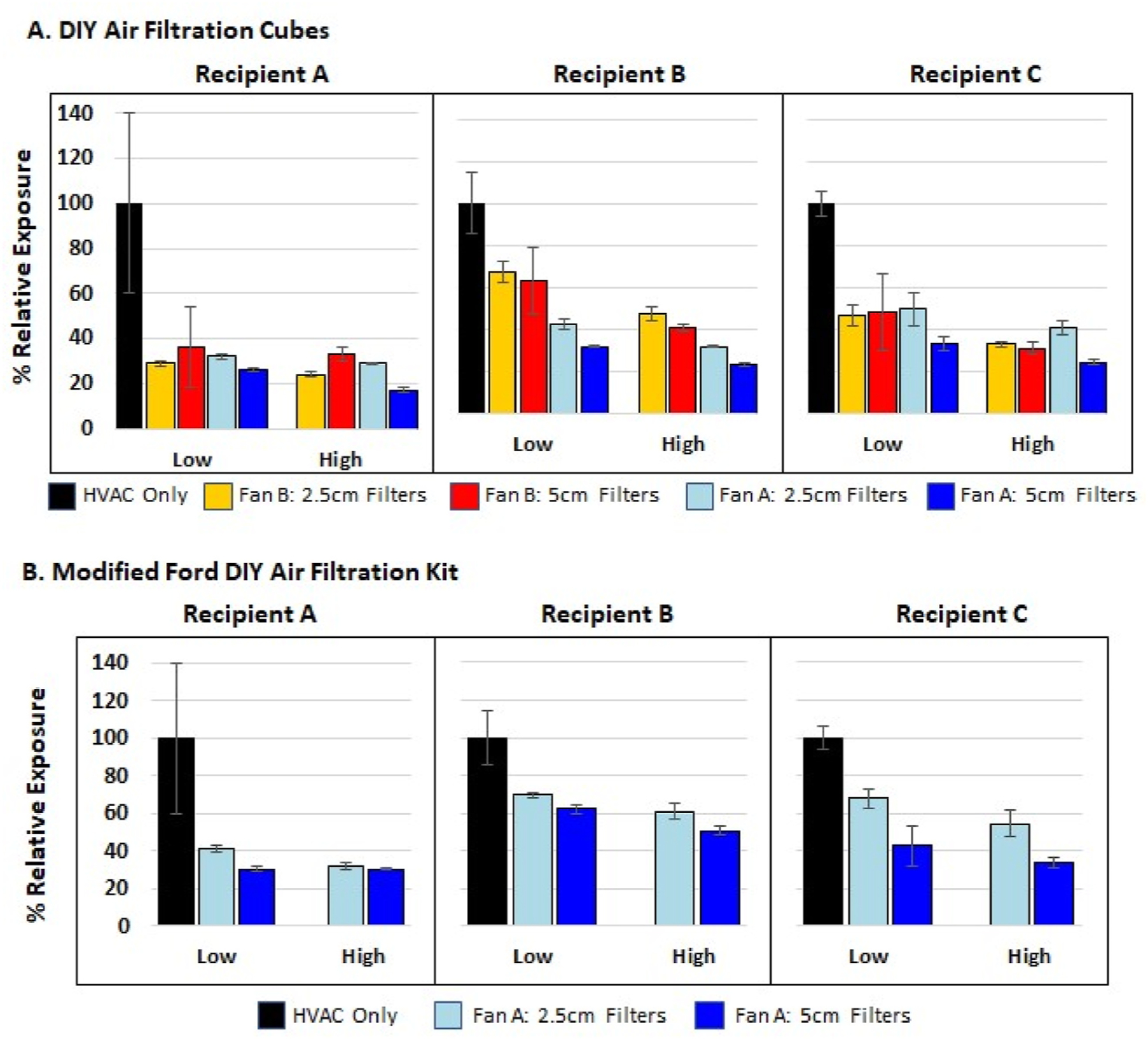
Effects of the type of DIY air filtration unit on relative exposure for each recipient. (A) HVAC only and two DIY air filtration cubes with 2.5 or 5 cm filters and either Fan B or A. (B) HVAC only and two modified Ford air filtration units with 2.5 or 5 cm filter and Fan A. For these experiments, one DIY air filtration unit was placed in the front and one in the back of the room, and both were operated at the same speed. The relative exposure for each recipient is normalized to the exposure measured when the HVAC system was operating at 2 ACH and no air filtration units were in use. The data are presented as the mean of four independent experiments with error bars representing one standard deviation. Bar mean and standard deviation values are presented in Supplemental Table S7.

The effects of the DIY air filtration cubes on the relative exposure of the recipient breathing simulators to simulated respiratory aerosols normalized to the room HVAC system set at 2 ACH are presented in Fig. 4A. Bars represent two DIY cubes, one in the front and one in the back of the room operating simultaneously. Averaging all three recipients, the DIY cubes with Fan B on high speed and 2.5 cm filters reduced relative exposure to 35%, while the DIY cubes with Fan A on high speed and 2.5 cm filters reduced relative exposure to 34%. Using 5 cm filters instead of 2.5 cm filters with Fan A on high speed reduced relative exposure by an additional 12% to give a relative aerosol exposure of 22% of the exposure seen with the HVAC system only.

The results from the modified Ford DIY air filtration units constructed with Fan A and with either 2.5 or 5 cm filters operating on low and high speeds are presented in Fig. 4B. Bars represent two DIY units, one in the front and one in the back of the room operating simultaneously. Fan B was not incorporated into the modified Ford DIY air filtration unit breathing experiments. Averaging across the three recipient locations, the relative exposure using the 2.5 cm filter was 59% on low speed and 49% on high. With the 5 cm filter, the relative exposure was 45% on low speed and 38% on high. Although the modified Ford DIY air filtration unit reduced exposure to below 60% for all combinations, when comparing the same parameters of filter thickness, fan model, and fan speed, the DIY cubes reduced the relative exposure approximately 20% more.

**Fig. 5.**
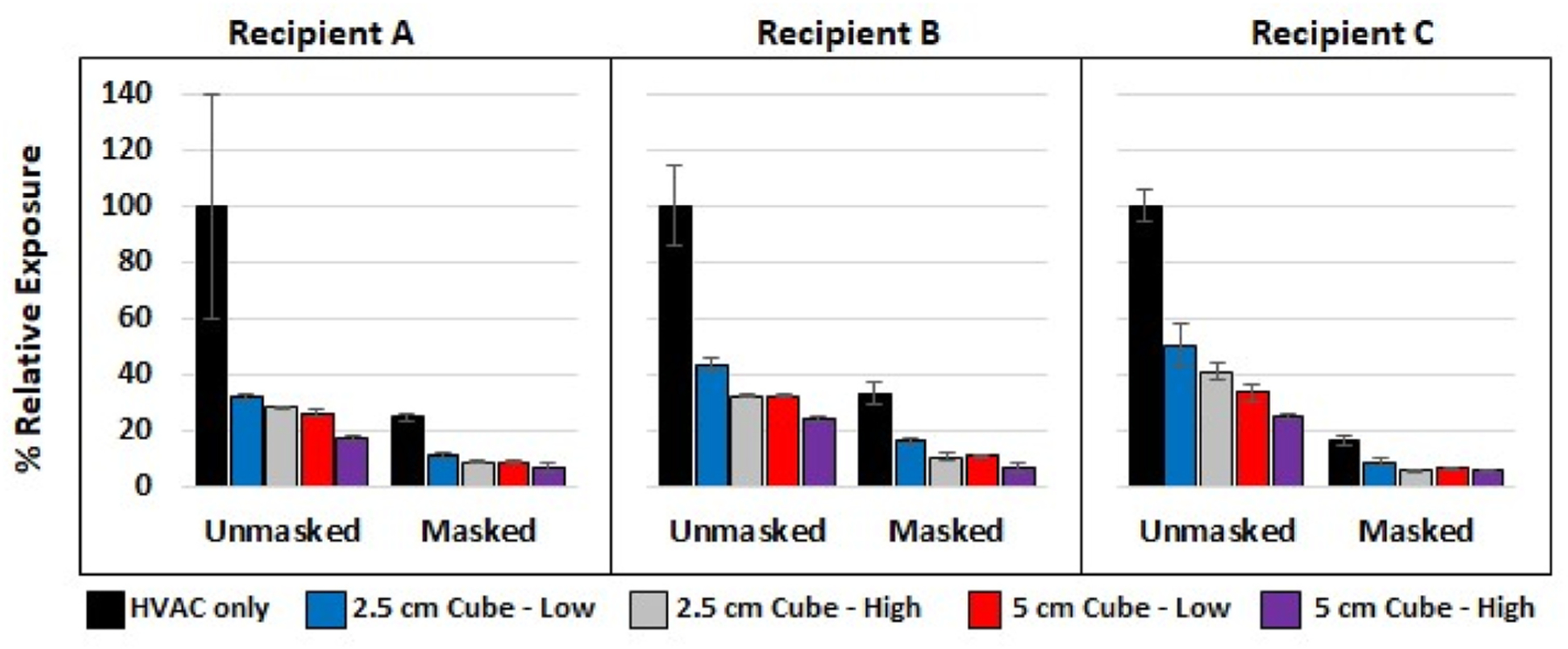
Effects of universal masking while operating two DIY air filtration cubes with Fan A, with one unit in the front and one in the back of the room. The relative exposure for each recipient is normalized to the exposure measured when the HVAC system was operating at 2 ACH and no air filtration units were in use. Fans were operated at both low and high speeds as shown. HVAC system was set at 2 ACH for all experiments. The data are presented as the mean of four independent experiments with error bars representing one standard deviation. Bar mean and standard deviation values are presented in Supplemental Table S7.

The effects of combining the use of face masks and the DIY cubes constructed with Fan A and either 2.5 cm or 5 cm filters is presented in Fig. 5. When DIY cubes were operated without masks on the recipients, the relative exposure was 41%–22% depending on filter thickness and fan speed. When the source and all recipients wore face masks (universal masking), the relative exposure for recipients with no DIY cubes operating was reduced to 25%. When recipients were universally masked and the DIY cubes were operating, the relative exposure was reduced to a minimum of 12% with 2.5 cm filters and the fan on low, and a minimum of 6% with the 5 cm filters and the fan on high when all three recipients were averaged. As would be expected, the overall reduction approximately equaled the product of the reduction from each strategy. For example, the 41% exposure with the DIY units on low multiplied by the 25% exposure with masking alone gives 10%, which is close to the 12% relative exposure seen when using both interventions. Similarly, the 22% relative exposure with the DIY units on high multiplied by 25% from masking gives 6%, which was very close to the observed results.

### 3.4. Regression model for masking and air change rate analysis

Two Multiple linear regression models were used to combine the results for all three recipients for a room average instead of examining each recipient individually since exposure varied between the recipients due to their physical location in the room. The first model examined the effects of universal masking and air exchange using the particle decay rates for each condition tested. A multiple linear regression model using the log-transformed mean mass concentration was used due to the violation of the constant variance assumption necessary for a linear model. Following the response transformation, the model passed the necessary assumptions of independence, normality, and constant variance with no outliers identified or removed. The overall fit of the model was considered good with a low residual standard error and an adjusted R^2^ = 0.937. The regression analysis for masking and effective air change rate is provided in Table 2A.

Universal masking alone without the use of the DIY units significantly reduced the proportional exposure to 0.30 (CI95%: 0.28–0.33; p < 0.001) of the exposure seen with no masking. Additionally, each increase of one effective ACH proportionally reduced the exposure to 0.90 (CI95%: 0.89–0.91; p < 0.001) based on log transformed data. The percentage relative exposure for each test condition can be determined by: Relative exposure = 100 × 0.30^^^(Masking) × 0.90^^^(ACH_eff_−1.89) where 0.30 represents the coefficient of proportional exposure remaining when masked versus no masking, 0.90 represents the proportion of exposure remaining per unit ACH increase, Masking are the coefficients of 0 for the no mask condition and 1 for the 3-ply cotton mask condition, ACH_eff_ is the effective air change rate for each test condition, and the 1.89 is the baseline air change rate of the room with the HVAC system set to 2 ACH.

### 3.5. Regression model for effective air change rate by DIY unit parameters

The second multiple linear regression model analyzed the effective air change rate by DIY unit parameters for DIY configuration, placement, filter width, fan model, and fan speed. The linear model passed the necessary assumptions of independence, normality, and constant variance with no outliers identified or removed. The second model did not require transformations. Overall fit of the model was considered good with low standard error and an adjusted R^2^ = 0.9356. The model base line was one modified Ford DIY air filtration unit with Fan B and a 2.5 cm filter, located in the back of the room with the fan speed on low. The regression analysis for effective air change rate by DIY parameters is provided in Table 2B.

Using the regression fit when only one unit was used in either the front or back of the room, no difference in relative exposure was seen due to unit location (0.02 ACH; CI95%: −0.56–0.60; p = 0.9525). When two units were operated concurrently, one in the front and one in the back of the room, the air change rate was increased by 4.81 ACH (CI95%: 4.27–5.34; p < 0.001) which was statistically significant compared with operating a single unit. Comparing the DIY configurations, two DIY cubes increased the effective air change rate by 5.12 ACH (CI95%: 4.71–5.52; p < 0.001) over two modified Ford DIY air filtration units. Constructing the Ford DIY units with Fan A increased the air change rate by 2.10 ACH (CI95%: 1.69–2.51; p < 0.001) compared with units constructed with Fan B. Units constructed with 5 cm filters showed an increase in the effective air change rate of 2.13 ACH (CI95%: 1.79–2.46; p < 0.001) compared with units constructed with 2.5 cm filters. The only DIY parameter that did not increase the air change rate by more than 2 ACH was fan speed. Fans operated on high speed increased the effective air change rate by 1.67 ACH (CI95%: 1.34–2.01; p < 0.001) which was still statistically significant compared with fans operated on low speed.

Based on the model fit, Fans A and B incorporated in the DIY filtration cubes resulted in a lower relative exposure for all the recipients compared with the modified Ford DIY air filtration units. The relative exposures for the DIY air filtration cube constructed with the 2.5 cm filters and Fan B were 63% on low speed and 49% on high, while the relative exposures using the DIY air filtration cubes with Fan A were 50% on low and 40% on high. When the DIY cubes were constructed with 5 cm filters, the DIY air filtration cube with Fan B reduced the relative exposure to 53% of the baseline on low and 44% on high, while the DIY air filtration cube with Fan A reduced exposure to 36% on low and 27% on high.

### 3.6. DIY unit position effects on aerosol exposure

**Fig. 6.**
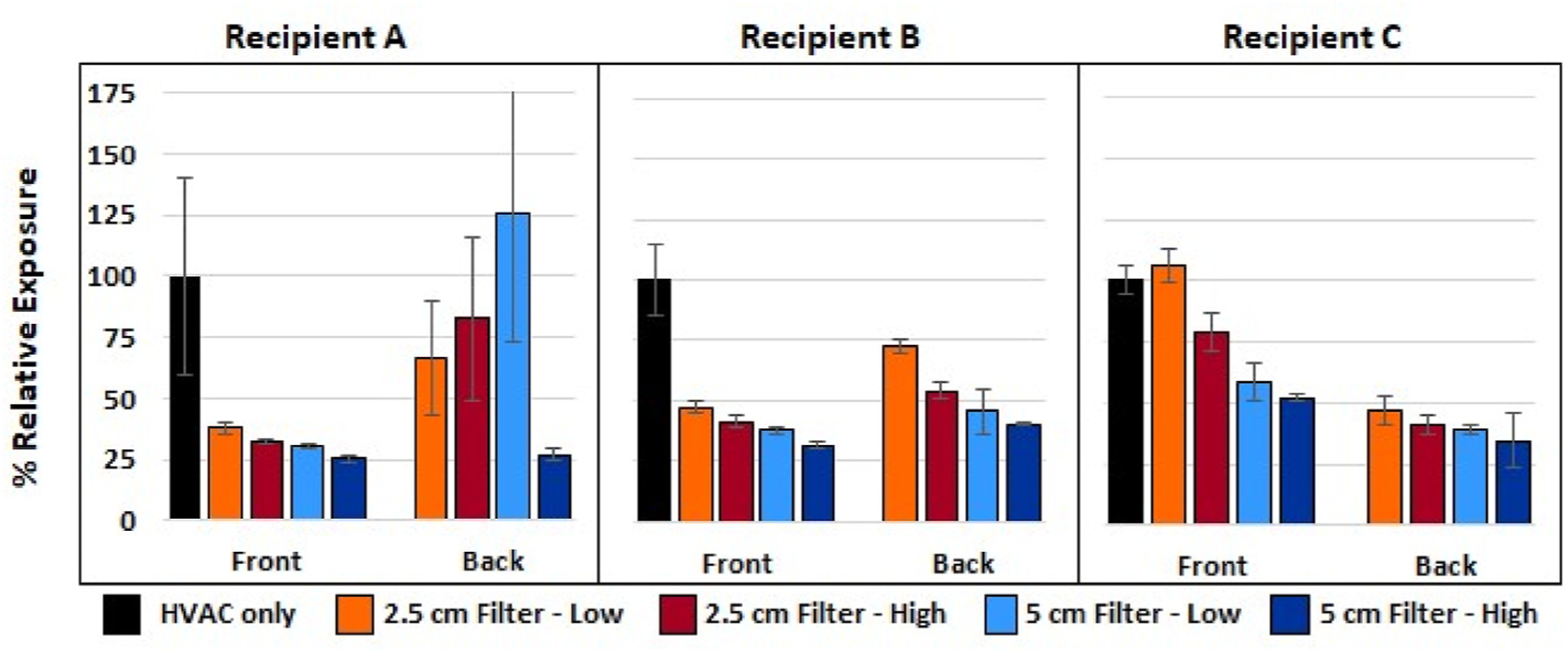
Location of one DIY cube unit and its effects on relative exposure for recipients. One DIY air filtration cube with either 2.5 or 5 cm filters and Fan A placed in the front or back of the room. The relative exposure for each recipient is normalized to the exposure measured when the HVAC system was operating at 2 ACH and no air filtration units were in use. The data are presented as the mean of four independent experiments with error bars representing one standard deviation. Bar mean and standard deviation values are presented in Supplemental Table S7.

The effects of DIY unit position on relative exposure were studied using DIY cubes constructed with either 2.5 or 5 cm filters and with Fan A, which was selected since it produced the highest airflow. The results of placing one DIY air filtration cube at the front or back of the room are presented in Fig. 6. With one unit at the front of the room, Recipient A had the lowest relative exposure of 31%, followed by Recipient B at 39%, and then Recipient C at 74%. With one unit at the front, air flowed towards Recipient C which transported the aerosol generated by the source toward Recipient C. Because of this air movement, in one case the relative exposure while using the 2.5 cm DIY cubes was marginally higher than when using the HVAC system alone. The opposite effect was observed when the unit was placed at the back of the room, with Recipient C having the lowest relative exposure of 40% followed by B at 53% and A at 75%. With the DIY unit at the back, the aerosol generated by the source was transported toward Recipient A causing it to have a higher relative exposure. This effect disappeared when two units were used as can be seen by comparing Fig. 6 (one unit) with Fig. 4A (two units). With one DIY unit in the front and a second in the back of the room, the relative exposure for Recipient A was 26%, Recipient B was 33% and Recipient C was 37%.

**Fig. 7.**
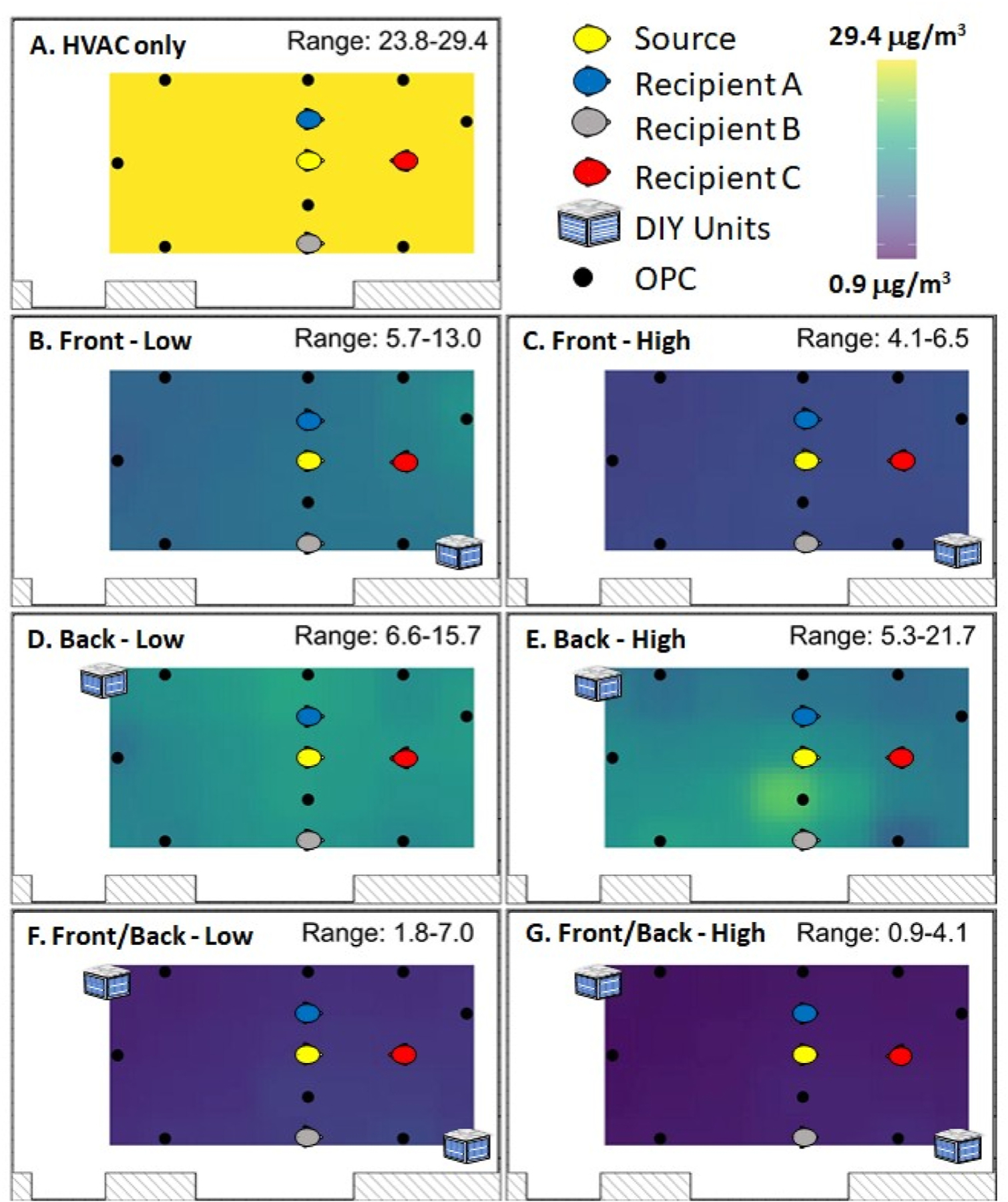
Spatial distribution of the mean aerosol mass concentration. The mean mass concentration measured by the OPC area samplers was quantified and overlain on the room diagrams. For these experiments, face masks were not worn by the simulators. The DIY air filtration cubes deployed were constructed with Fan A and 5 cm filters. (A) HVAC system set at 2 ACH without DIY air filtration cubes; (B) one DIY cube placed in the front of the room with the fan speed on low; (C) one DIY cube placed in the front with the fan speed on high; (D) one DIY cube placed in the back with the fan speed on low; (E) one DIY cube placed in the back with the fan speed on high; (F) two DIY cubes placed with one in front and one in back and with the fan speeds on low; and (G) two DIY cubes placed with one in front and one in back and with the fan speeds on high. The coloration has been normalized to the concentration range observed among all trials, with purple as the lowest area concentration and yellow as the highest. (For interpretation of the references to color in this figure legend, the reader is referred to the Web version of this article.)

To confirm directional airflow movement of the aerosol in the room, the OPC data are presented as a spatial mean mass concentration (μg/m^3^) distribution (Fig. 7) with the DIY air filtration cube constructed with Fan A and 5 cm filters. Using the HVAC system only, the concentration ranged from 23.8 to 29.4 μg/m^3^ and was evenly distributed in the room (Fig. 7A). When one unit was placed at the front of the room and on low speed, the concentration was highest towards the front of the room at 13.0 μg/m^3^ compared with the back at 5.7 μg/m^3^ (Fig. 7B). With the DIY cube at the same location but on high speed (Fig. 7C), the overall concentration was lower with a tighter range (front 6.5 μg/m^3^ and back 4.1 μg/m^3^). When the unit was moved to the back of the room, the highest concentration occurred toward Recipient A when on low speed (Fig. 7D) and toward Recipient B when on high speed (Fig. 7E). When two units were deployed, one in front and one in back, the overall concentration was lower with a smaller spatial variation in the range of aerosol concentrations compared with using one unit. The concentration for two units on low speed was between 1.8 and 7.0 μg/m^3^ (Fig. 7F) and on high was between 0.9 and 4.1 μg/m^3^ (Fig. 7G).

A second spatial distribution (Figure S2) is in the Supplemental Material and is colorized for each panels unique range. It additionally shows how the DIY units affected the particle distribution in the room, however it’s difficult to compare panels since each panel is unique.

## 4. Discussion

This investigation demonstrated that several significant factors affect the reduction in aerosol exposure produced using DIY air filtration units. First, increasing the MERV 13 filter thickness, which is directly related to the surface area of the filters, resulted in significantly lower aerosol exposures for both types of DIY air filtration units. The 5 cm filters have more than twice the surface area of the 2.5 cm filters (Supplemental Table S1). As shown by the airflow measurements, increasing the surface area of a filter reduces the flow resistance and allows for more airflow through the filter, thereby filtering more air. While the modified Ford DIY unit can use thicker filters relatively easily, using filters thicker than 5 cm in the DIY cubes would require the filter frame to overlap filter media during construction or would require a more complex design to avoid overlapping, thus diminishing the construction simplicity of the unit. It should be noted that the original Ford DIY air filtration units used 10 cm thick filters rather than the 2.5 and 5 cm used in our modified design; thus, the original design likely would have provided better performance than our modified version. The thinner filters which are widely available commercially were used in this study to represent filters that would be typically used by the public to construct DIY units.

Second, the number of filters in the design of the units had a significant impact on aerosol reduction, and this reduction also can be attributed to the increase in the total surface area of the filters. The DIY air filtration cube with four 5 cm filters had a combined surface area of 4.52 m^2^ compared with 1.13 m^2^ for the modified Ford DIY air filtration unit with a single 5 cm filter, and the DIY cube consequently was better at reducing exposure among the recipients. As with increasing the filter depth, increasing the number of filters increased the filter surface area which allowed more air to be filtered and increased the effective air change rate.

Third, the fan airflow rate had a significant effect on exposure reduction. Fan A which had a higher airflow rate, consistently out-performed Fan B. Unfortunately, only a few fan manufacturers provide fan specifications including airflow rates on their products, making it difficult to determine which fans to use when constructing a DIY air filtration unit. In our study, only one out of seven fans purchased provided flow rate information and the rate was determined by the Air Movement and Control Association (AMCA) standard 230–99 [47], which is primarily for ceiling fans and involves measuring the thrust of the fan. The AMCA standard flow rate for Fan A was much higher (1463 CFM with the fan on low and 2163 CFM on high) than the flow rate obtained in this study with the Alnor Balometer (712 CFM on low and 959 CFM on high).

In addition to increasing the filtration rate of the room air, using two DIY units instead of one also increased air mixing and produced a more uniform reduction in aerosol concentration. When a single DIY air filtration cube was placed at the front or back of the room, the cube substantially changed the airflow pattern in the room, causing some recipients to have a higher relative exposure. When a second unit was added to the room, exposure for all recipients was lower and the observed aerosol distribution was more consistent throughout the room. A similar pattern was observed in a previous study with commercially available portable HEPA air cleaners [21]. These results suggest that operating two or more air cleaning units in the room rather than a single unit provides better air mixing and a more even reduction in aerosol concentration, which reduces the possibility of pulling virus laden air toward the occupants in a room. If possible before adding any air filtration unit to a room, a comprehensive evaluation of the room airflow should be conducted to examine how an air cleaning device might influence airflow to avoid unintended consequences.

An important consideration when using a DIY air filtration unit in a classroom setting is the noise level. Unfortunately, fans with higher flow rates and on higher settings tend to be noisier. When single units were operated on low speed, 5 out of 7 fans were below the ANSI maximum background noise level of 50 dB, while only one unit was below the limit on high speed. As stated above, the best practice for aerosol reduction is operating two or more units simultaneously in a room. When operating two units simultaneously with either Fan A or B, only Fan B operating on low was below the maximum suggested background level, and when operating on high it was 2–4 dB above the maximum level depending on filter width. If noise was the most important issue, then the DIY air filtration cube constructed with Fan B operating on low would be the best selection. However, based on the regression analysis that would provide a 53% relative exposure which is significantly higher than the 27% relative exposure seen when using a cube with Fan A on high. Thus, when selecting a fan for a DIY air filtration unit, tradeoffs are necessary between airflow rates, noise levels, power consumption, cost, and availability, and no particular fan model will be the best choice for all situations.

Currently there are no standards for constructing DIY units, which has led to several variations and designs that can significantly influence their effectiveness. Many filter models are available commercially with a wide range of filtration efficiencies, and with no standard filter recommendations, units constructed by the public will inevitably vary greatly in performance. Filters are available in different sizes and thickness, resulting in additional variation as demonstrated in this study. Anecdotal evidence suggests that 2.5 cm filters are the most common thickness of filter in home HVAC systems and thus would likely be the most common filters used to build DIY units. This would result in higher relative exposures than units constructed with 5 cm filters. Our study only incorporated one brand of MERV 13 filters and did not examine lower or higher rated filters or different brands. Additionally, several brands of fans are available commercially with unreported airflow rates, which would contribute to a wide range of DIY unit efficiencies. The number of filters used in construction also will impact efficiency. This study used four filters to construct the DIY air filtration cubes which performed significantly better than the one filter modified Ford DIY air filtration unit. Not examined was the five-filter design of the DIY air filtration cube which may have a higher efficiency since it can theoretically draw more air. The age of the fan used to build a DIY unit might impact efficiency and fans manufactured prior to 2012 may not have safety features to reduce the risk of the fan overheating [48]. Finally, in order to help increase the airflow through the filters and prevent air from bypassing the filters, the filter-to-filter and fan-to-filter joints should be completely covered, all fan chase holes should be sealed, and a fan shroud should be added.

Most DIY units are constructed by individuals and thus are not subject to the quality control measures typically used by a commercial manufacturer, which can lead to issues that could degrade performance. In addition, testing DIY air filtration units for proper performance requires specialized equipment and knowledge that are not widely available. Thus, unfortunately, the typical person building their own air filtration unit does not have a ready way to test the unit after construction to verify that it is working as intended. One possible solution to address the construction and performance variability of DIY air filtration units would be to develop a test method using low-cost commercially available air quality monitors (PM2.5 monitors) that measure airborne particulate matter. However, further research is needed in this area.

In addition to the use of portable air filtration units, other measures such as the universal use of face masks, physical distancing, and reducing indoor space occupancy can also reduce exposure to potentially infectious aerosol particles, and these measures work best when used in combination in a multi-layer approach. In our study, universal masking with a 3-ply cotton mask reduced exposure by 70%, which was consistent with previous studies [21]. More importantly, when universal masking was combined with the DIY air filtration cubes, exposure was reduced by 88–94% depending on filters and fan speed. These results support the concept of multiple mitigation strategies as the best way at reducing relative exposure to infectious aerosols.

Finally, the study design of our investigation had several limitations. First, the source and participant simulators were unlike humans in some important respects. The simulators were static and did not move around the room, did not contain a heated body source, and did not exhale breaths of warm and humid air that would create a thermal plume, all of which could influence the airflow pattern and aerosol dispersion for the recipients. Second, this investigation was conducted in a single classroom-style room with a unique airflow pattern. The results in this room and the placement of the DIY air filtration units would not necessarily transfer to another room and would be dependent on the airflow in a particular room. Third, the room did not contain furniture which would affect the flow pattern and could have resulted in dead spaces for aerosols to accumulate. Fourth, the study looked at a limited size range of particles from 0.3 to 3 μm, which is the size range of bioaerosol particles that remain airborne for longer time but are large enough to carry pathogens. However, humans do produce particles across a much broader size range [49,50].

## 5. Conclusions

The DIY air filtration units reduced aerosol exposure up to 73% depending on the design, filter thickness, and fan airflow. In general, our results show that the performance of the DIY air filtration units is largely a function of the total airflow rate through the filters. Constructing DIY air filtration units with more filters (DIY cube), thicker filters (which have a greater surface area), and a higher airflow fan, and the use of two DIY units instead of one, all added 2 or more effective ACH to the overall room air change rate, which led to a corresponding reduction in aerosol concentration. However, the amount of variability in performance seen with different DIY configurations, fans, and filters shows that DIY air filtration units must be used with caution since individual units’ performance will be unique. In addition, potential problems with construction quality such as leaks and gaps could substantially affect the performance of DIY air filtration units. Unfortunately, there is at present no simple way for the do-it-yourselfer to verify that their DIY unit is performing as expected. DIY air filtration units may be effective for temporary use until commercial portable air cleaners with known performance characteristics can be secured or used in areas that cannot obtain portable air cleaners. However, the EPA does not recommend the DIY units as a permanent alternative to products of known performance (such as commercially available portable air cleaners) [48].

## Supplementary Material

supplemental

## Figures and Tables

**Table 1:** Airflow Measurements. Fan airflow measured for a single unit at indicated fan speed with an Alnor Balometer. Unit of measurement is cubic feet per minute (CFM) and the values are an n = 1. MOD Ford = Modified Ford DIY air filtration unit. DIY Cube = DIY air filtration cube.

Speed	Shrouded		MOD Ford with 2.5 cm Filter		MOD Ford with 5 cm Filter		DIY Cube with 2.5 cm Filter		DIY Cube with 5 cm Filter	
	Low	High	Low	High	Low	High	Low	High	Low	High
Fan A	712	959	129	197	312	450	395	564	560	730
Fan B	428	625	104	185	172	281	247	410	296	504
Fan C	494	667	160	268	223	350	282	465	354	554
Fan D	478	650	174	263	234	344	314	462	412	553
Fan E	503	863	128	264	180	334	264	518	335	676
Fan F	622	920	122	250	228	429	358	616	448	737
Fan G	450	645	150	247	201	313	261	437	342	538

**Table 2:** Regression analysis.

Regression analysis of mean mass concentration by effective ACH and use of face masks						
	Regression Coefficients^a^		Back-Transformed^a^			

Parameter	Estimate	CI95%^b^	Estimate	CI95%	t-value	p-value
Intercept	3.02	2.94 to 3.10	20.47	18.84 to 22.24	72.24	<0.0001
Condition: Masked	−1.19	−1.28 to −1.10	0.30	0.28 to 0.33	−26.96	<0.0001
Effective ACH	−0.11	−0.12 to −0.10	0.90	0.89 to 0.91	−20.02	<0.0001
Regression analysis of effective ACH by DIY unit parameters						
Parameter	Estimate		Standard Error	CI95%	t-value	p-value

Intercept	−3.17		0.37	−3.91 to −2.43	−8.57	<0.001
Fan model: Fan A	2.10		0.21	1.69 to 2.51	10.23	<0.001
Fan Speed: High	1.67		0.17	1.34 to 2.01	10.00	<0.001
Placement: Front	0.02		0.29	−0.56 to 0.60	0.06	0.9525
Placement: Front and Back	4.81		0.27	4.27 to 5.34	17.96	<0.001
Filter Width: 5 cm	2.13		0.17	1.79 to 2.46	12.70	<0.001
DIY Design: Cube	5.12		0.20	4.71 to 5.52	24.97	<0.001

Baseline condition for regression analysis was Fan B (fan model), Low fan speed, Back placement, 2.5 cm filter width, and a Ford DIY design.

Adj. R2 = 0.9356.

Baseline condition for regression analysis was 1.89 ACH (HVAC set at 2 ACH and no DIY units) and Unmasked simulators.

Adj. R2 = 0.9370.

a The Estimate and CI95% Regression Coefficients were derived from a multiple linear regression model using log-transformed exposure concentration. The Intercept back-transformed Estimate and CI95% denote proportion of exposure remaining per unit variable increase of Condition and Effective ACH, while the Intercept denotes the baseline aerosol exposure concentration in μg/m^3^.

b 95% Confidence Interval of the Estimate.

## Data Availability

Data will be made available on request.
