# Supplementary material for: Efficacy of Do-It-Yourself air filtration units in reducing exposure to simulated respiratory aerosols
Source: Build Environ. Author manuscript; Available in PMC 2022 Dec 19. (PMC9759459; doi:10.1016/j.buildenv.2022.109920)
Supplement: supplemental [file NIHMS1870659-supplement-supplemental.docx]

**Supplemental Material**

**Room Environmental Conditions and Mask Fit**

The follow conditions in the room were across all experiments:

Ambient temperature 22.2 °C (SD 1.1 °C)

Relative humidity 48.4% (SD 7.9%)

Barometric pressure 98.05 kPa (SD 0.65 kPa).

The following were mean fit factor for the manikins:

Source: 1.4 (SD 0.2).

Recipient A: 3.2 (SD 0.5)

Recipient B: 3.2 (SD 0.6)

Recipient C: 3.4 (SD 0.2)

**Supplemental Table S1**. Filter technical specifications provided by W.W. Grainger.

|  | Air Handler 1” (2.5 cm) Filter ^a^ | Air Handler 2” (5 cm) Filter ^b^ |
| --- | --- | --- |
| Mfr. Model # | 11C876 | 2EKJ5 |
| Performance Rating | MERV 13 | MERV 13 |
| Style | Pleated, Panel | Pleated, Panel |
| Frame or Header Material | Beverage Board | Beverage Board |
| Media Material | Synthetic | Synthetic |
| Nominal Filter Size | 20” x 20” x 1” | 20” x 20” x 2” |
| Actual Filter Size | 19.5” x 19.5” x 0.75” | 19.5” x 19.5” x 1.75” |
| Media Area | 5.1 sq ft (0.47 m^2^) | 12.2 sq ft (1.13 m^2^) |
| Filters Out | Cooking Oil, Copier Toner, Droplet Nuclei (Sneeze), Insecticide Dust, MERV 13-16 All Bacteria, Most Face Powder, Most Smoke, Paint Pigments | Cooking Oil, Copier Toner, Droplet Nuclei (Sneeze), Insecticide Dust, MERV 13-16 All Bacteria, Most Face Powder, Most Smoke, Paint Pigments |
| Max. Velocity | 500 fpm | 500 fpm |
| Removes Particles Down to | 1.0 to 3.0 micron | 1.0 to 3.0 micron |
| Airflow @300 fpm | 833 cfm | 833 cfm |
| Airflow @500 fpm | 1,389 cfm | 1,389 cfm |
| Initial Resistance @ 300 fpm | 0.22 in wc | 0.22 in wc |
| Initial Resistance @ 500 fpm | 0.37 in wc | 0.37 in wc |
| Final Recommended Resistance | 1.0 in wc | 1.0 in wc |
| Standards | UL 900 Classified | UL 900 Classified |

^a^ W.W. Grainger, LEED/Green Pleated Air Filter, 20x20x1, MERV 13, 2022. <https://www.grainger.com/product/AIR-HANDLER-LEED-Green-Pleated-Air-Filter-11C876>. (Accessed 4/11/2022.

^b^ W.W. Grainger, LEED/Green Pleated Air Filter, 20x20x2, MERV 13, 2022. <https://www.grainger.com/product/AIR-HANDLER-LEED-Green-Pleated-Air-Filter-2EKJ5>. (Accessed 4/11/2022.

**Supplemental Table S2**. Equivalent continuous sound pressure level (L_eq_) for 5 seconds measured using the decibel A (dBA) scale. Measurements were acquired at the eight OPC locations and then averaged for a mean room noise level for each fan and DIY unit. Measurements were obtained with fans and units running at the indicated speed and room HVAC set at 2 ACH. Values are reported as a mean dBA with one standard deviation The background room mean decibel level measured at the eight OPC locations was 36.1 ± 0.5 dBA with the HVAC set at 2 ACH. MOD Ford = Modified DIY Ford Air filtration unit. DIY Cube = DIY air filtration cube.

| Fan Speed Low | Shrouded | MOD Ford with 2.5 cm Filter | MOD Ford with 5 cm Filter | DIY Cube with 2.5 cm Filter | DIY Cube with 5 cm Filter |
| --- | --- | --- | --- | --- | --- |
| Fan A | 55.8 ± 2.1 | 57.1 ± 2.1 | 57.1 ± 2.1 | 57.0 ± 2.4 | 56.2 ± 2.2 |
| Fan B | 41.3 ± 1.7 | 40.6 ± 1.6 | 40.5 ± 1.7 | 39.9 ± 1.8 | 39.7 ± 1.7 |
| Fan C | 50.3 ± 2.0 | 48.1 ± 1.9 | 48.9 ± 2.1 | 48.2 ± 2.1 | 48.1 ± 2.0 |
| Fan D | 52.6 ± 2.1 | 51.7 ± 2.2 | 52.0 ± 2.2 | 52.3 ± 2.3 | 52.5 ± 2.2 |
| Fan E | 47.7 ± 3.4 | 43.9 ± 1.8 | 43.5 ± 1.9 | 44.8 ± 2.1 | 43.8 ± 1.8 |
| Fan F | 45.6 ± 2.0 | 45.5 ± 2.0 | 46.2 ± 1.9 | 45.3 ± 2.0 | 45.4 ± 2.2 |
| Fan G | 49.0 ± 2.0 | 48.9 ± 2.2 | 48.7 ± 2.2 | 47.0 ± 1.8 | 47.8 ± 2.1 |
| Fan A (1 front/1 back) | 58.8 ± 1.0 | 61.0 ± 1.3 | 59.8 ± 1.1 | 60.0 ± 1.1 | 58.8 ± 0.8 |
| Fan B (1 front/1 back) | 45.9 ± 1.0 | 44.3 ± 1.0 | 44.5 ± 1.0 | 46.6 ± 1.3 | 43.4 ± 1.1 |
| Fan Speed High |  |  |  |  |  |
| Fan A | 62.2 ± 2.3 | 63.8 ± 2.4 | 63.5 ± 2.3 | 62.5 ± 2.4 | 62.3 ± 2.3 |
| Fan B | 50.5 ± 1.7 | 50.4 ± 2.2 | 48.7 ± 1.9 | 48.6 ± 2.1 | 50.4 ± 2.5 |
| Fan C | 58.2 ± 2.3 | 57.2 ± 2.2 | 57.7 ± 2.2 | 57.5 ± 2.2 | 57.0 ± 2.3 |
| Fan D | 59.6 ± 2.4 | 58.4 ± 2.4 | 58.7 ± 2.4 | 59.2 ± 2.3 | 59.2 ± 2.3 |
| Fan E | 57.5 ± 2.4 | 54.4 ± 2.2 | 54.9 ± 2.1 | 55.5 ± 2.3 | 56.8 ± 2.5 |
| Fan F | 55.2 ± 2.5 | 55.7 ± 2.3 | 55.1 ± 2.2 | 54.8 ± 2.1 | 55.4 ± 2.3 |
| Fan G | 57.6 ± 2.4 | 56.9 ± 2.3 | 57.4 ± 2.3 | 56.0 ± 2.3 | 57.0 ± 2.2 |
| Fan A (1 front/1 back) | 65.6 ± 1.0 | 66.4 ± 1.2 | 66.3 ± 1.0 | 65.8 ± 1.2 | 65.5 ± 1.1 |
| Fan B (1 front/1 back) | 55.1 ± 1.0 | 53.3 ± 1.1 | 54.0 ± 1.1 | 52.5 ± 1.1 | 54.3 ± 1.1 |

**Supplemental Table S3**. Fan current (Amperes) measured with a Voltage multimeter. MOD Ford = Modified Ford DIY air filtration unit. DIY Cube = DIY air filtration cube.

|  | Shrouded only | | DIY Cube with 2.5 cm Filter | | DIY Cube with 5 cm Filter | | MOD Ford with 2.5 cm Filter | | MOD Ford with 5 cm Filter | |
| --- | --- | --- | --- | --- | --- | --- | --- | --- | --- | --- |
| Fan Speed | Low | High | Low | High | Low | High | Low | High | Low | High |
| Fan A | 0.611 | 0.877 | 0.598 | 0.859 | 0.596 | 0.866 | 0.593 | 0.847 | 0.614 | 0.896 |
| Fan B | 0.494 | 0.652 | 0.477 | 0.634 | 0.481 | 0.647 | 0.485 | 0.646 | 0.477 | 0.626 |
| Fan C | 0.353 | 0.466 | 0.354 | 0.468 | 0.352 | 0.467 | 0.356 | 0.477 | 0.355 | 0.470 |
| Fan D | 0.354 | 0.458 | 0.361 | 0.468 | 0.360 | 0.464 | 0.355 | 0.462 | 0.355 | 0.460 |
| Fan E | 0.588 | 0.772 | 0.578 | 0.779 | 0.587 | 0.784 | 0.581 | 0.766 | 0.584 | 0.770 |
| Fan F | 0.626 | 0.913 | 0.627 | 0.933 | 0.624 | 0.934 | 0.623 | 0.904 | 0.627 | 0.933 |
| Fan G | 0.351 | 0.461 | 0.350 | 0.457 | 0.353 | 0.461 | 0.350 | 0.462 | 0.350 | 0.458 |

**Supplemental Table S4**. Fan power (Watts) calculated using the Watts Law Formula. Outlet voltage used by all fans measured 120 Volts. Current measurements for each fan are listed in supplemental table S3. MOD Ford = Modified Ford DIY air filtration unit. DIY Cube = DIY air filtration cube.

|  | Shrouded only | | DIY Cube with 2.5 cm Filter | | DIY Cube with 5 cm Filter | | MOD Ford with 2.5 cm Filter | | MOD Ford with 5 cm Filter | |
| --- | --- | --- | --- | --- | --- | --- | --- | --- | --- | --- |
| Fan Speed | Low | High | Low | High | Low | High | Low | High | Low | High |
| Fan A | 70.56 | 102.12 | 70.32 | 101.64 | 70.08 | 102.48 | 69.72 | 100.20 | 72.24 | 106.08 |
| Fan B | 57.84 | 76.80 | 55.80 | 74.64 | 56.28 | 76.20 | 56.76 | 76.08 | 55.80 | 73.68 |
| Fan C | 40.92 | 54.48 | 41.04 | 54.72 | 40.80 | 54.60 | 41.28 | 55.80 | 41.16 | 54.96 |
| Fan D | 41.04 | 53.52 | 41.88 | 54.72 | 41.76 | 54.24 | 41.16 | 54.00 | 41.16 | 53.76 |
| Fan E | 69.12 | 91.20 | 67.92 | 92.04 | 69.00 | 92.64 | 68.28 | 90.48 | 68.64 | 90.96 |
| Fan F | 73.68 | 108.12 | 73.80 | 110.52 | 73.44 | 110.64 | 73.32 | 107.04 | 73.80 | 110.52 |
| Fan G | 40.68 | 53.88 | 40.56 | 53.40 | 40.92 | 53.88 | 40.56 | 54.00 | 40.56 | 53.52 |

**Supplemental Table S5**. Fan blade RPM. MOD Ford = Modified Ford DIY air filtration unit. DIY Cube = DIY air filtration cube.

|  | Shrouded only | | DIY Cube with 2.5 cm Filter | | DIY Cube with 5 cm Filter | | MOD Ford with 2.5 cm Filter | | MOD Ford with 5 cm Filter | |
| --- | --- | --- | --- | --- | --- | --- | --- | --- | --- | --- |
| Fan Speed | Low | High | Low | High | Low | High | Low | High | Low | High |
| Fan A | 953 | 1250 | 1052 | 1310 | 985 | 1268 | 1051 | 1290 | 920 | 1189 |
| Fan B | 602 | 878 | 579 | 870 | 597 | 907 | 612 | 860 | 592 | 821 |
| Fan C | 911 | 1202 | 805 | 1148 | 813 | 1166 | 766 | 1097 | 819 | 1113 |
| Fan D | 960 | 1223 | 908 | 1236 | 978 | 1268 | 906 | 1189 | 909 | 1196 |
| Fan E | 592 | 1010 | 540 | 930 | 587 | 1000 | 596 | 906 | 557 | 875 |
| Fan F | 610 | 1121 | 567 | 852 | 584 | 870 | 653 | 913 | 600 | 837 |
| Fan G | 803 | 1172 | 790 | 1143 | 816 | 1160 | 808 | 1105 | 819 | 1116 |

**Table S6**. Total air change rate and estimated CADR. The ACH values shown for the DIY units reflect the combination of the room ventilation by the HVAC system and the filtration effect of two DIY units operating simultaneously, one in the front and one in the back of the room. For all experiments, the room HVAC system was operating at a nominal setting of 2 ACH (actual rate 1.89 ACH). Values represents the mean and standard deviation of a minimum of three independent measurements.

| Fan | Filter Width | DIY Number and Type | Fan Speed | Total Effective ACH | Standard Deviation | ACH achieved by DIY units | Estimated CADR |
| --- | --- | --- | --- | --- | --- | --- | --- |
| Fan A | 1 | 1 Cube | Low | 4.79 | 0.05 | 2.90 | 308 |
| Fan A | 1 | 1 Cube | High | 5.64 | 0.04 | 3.75 | 397 |
| Fan A | 2 | 1 Cube | Low | 6.18 | 0.02 | 4.29 | 455 |
| Fan A | 2 | 1 Cube | High | 7.22 | 0.08 | 5.33 | 565 |
| Fan A | 1 | 2 Cubes | Low | 8.44 | 0.24 | 6.55 | 695 |
| Fan A | 1 | 2 Cubes | High | 10.59 | 0.11 | 8.70 | 922 |
| Fan A | 2 | 2 Cubes | Low | 11.54 | 0.01 | 9.65 | 1023 |
| Fan A | 2 | 2 Cubes | High | 14.26 | 0.23 | 12.37 | 1311 |
| Fan B | 1 | 2 Cubes | Low | 6.23 | 0.26 | 4.34 | 460 |
| Fan B | 1 | 2 Cubes | High | 8.67 | 0.10 | 6.78 | 719 |
| Fan B | 2 | 2 Cubes | Low | 7.91 | 0.07 | 6.02 | 638 |
| Fan B | 2 | 2 Cubes | High | 9.72 | 0.19 | 7.83 | 830 |
| Fan A | 1 | 2 Ford Units | Low | 3.08 | 0.01 | 1.19 | 126 |
| Fan A | 1 | 2 Ford Units | High | 4.38 | 0.19 | 2.49 | 264 |
| Fan A | 2 | 2 Ford Units | Low | 5.10 | 0.22 | 3.21 | 340 |
| Fan A | 2 | 2 Ford Units | High | 8.17 | 0.10 | 6.28 | 666 |
| Fan B | 1 | 2 Ford Units | Low | 2.64 | 0.01 | 0.75 | 79 |
| Fan B | 1 | 2 Ford Units | High | 3.44 | 0.06 | 1.55 | 164 |
| Fan B | 2 | 2 Ford Units | Low | 4.10 | 0.10 | 2.21 | 234 |
| Fan B | 2 | 2 Ford Units | High | 5.84 | 0.10 | 3.95 | 418 |

**Supplemental Table S7.** Mean and standard deviation values for figures 4-6.

| **Figure 4A** | | | | | | |  |
| --- | --- | --- | --- | --- | --- | --- | --- |
| Breather | Fan Model | Fan Speed | Filter Width (cm) | DIY Configuration | Normalized Mean (%) | Standard Deviation |  |
| Recipient A | No Unit | None | None | None | 1.00 | 0.40 |  |
| Recipient A | Fan B | Low | 2.5 | 2-Cube | 0.29 | 0.01 |  |
| Recipient A | Fan B | Low | 5 | 2-Cube | 0.36 | 0.18 |  |
| Recipient A | Fan A | Low | 2.5 | 2-Cube | 0.32 | 0.01 |  |
| Recipient A | Fan A | Low | 5 | 2-Cube | 0.26 | 0.01 |  |
| Recipient A | Fan B | High | 2.5 | 2-Cube | 0.24 | 0.01 |  |
| Recipient A | Fan B | High | 5 | 2-Cube | 0.23 | 0.03 |  |
| Recipient A | Fan A | High | 2.5 | 2-Cube | 0.29 | 0.00 |  |
| Recipient A | Fan A | High | 5 | 2-Cube | 0.17 | 0.01 |  |
| Recipient B | No Unit | None | None | None | 1.00 | 0.15 |  |
| Recipient B | Fan B | Low | 2.5 | 2-Cube | 0.68 | 0.05 |  |
| Recipient B | Fan B | Low | 5 | 2-Cube | 0.64 | 0.16 |  |
| Recipient B | Fan A | Low | 2.5 | 2-Cube | 0.43 | 0.03 |  |
| Recipient B | Fan A | Low | 5 | 2-Cube | 0.32 | 0.01 |  |
| Recipient B | Fan B | High | 2.5 | 2-Cube | 0.47 | 0.03 |  |
| Recipient B | Fan B | High | 5 | 2-Cube | 0.41 | 0.01 |  |
| Recipient B | Fan A | High | 2.5 | 2-Cube | 0.32 | 0.01 |  |
| Recipient B | Fan A | High | 5 | 2-Cube | 0.24 | 0.01 |  |
| Recipient C | No Unit | None | None | None | 1.00 | 0.06 |  |
| Recipient C | Fan B | Low | 2.5 | 2-Cube | 0.47 | 0.05 |  |
| Recipient C | Fan B | Low | 5 | 2-Cube | 0.48 | 0.18 |  |
| Recipient C | Fan A | Low | 2.5 | 2-Cube | 0.50 | 0.08 |  |
| Recipient C | Fan A | Low | 5 | 2-Cube | 0.33 | 0.03 |  |
| Recipient C | Fan B | High | 2.5 | 2-Cube | 0.33 | 0.02 |  |
| Recipient C | Fan B | High | 5 | 2-Cube | 0.31 | 0.03 |  |
| Recipient C | Fan A | High | 2.5 | 2-Cube | 0.41 | 0.03 |  |
| Recipient C | Fan A | High | 5 | 2-Cube | 0.25 | 0.01 |  |
|  |  |  |  |  |  |  |  |
| **Figure 4B** | | | | | | |  |
| Breather | Fan Model | Fan Speed | Filter Width (cm) | DIY Configuration | Normalized Mean (%) | Standard Deviation |  |
| Recipient A | None | None | None | None | 1.00 | 0.40 |  |
| Recipient A | Fan A | Low | 2.5 | 2-Ford | 0.41 | 0.02 |  |
| Recipient A | Fan A | Low | 5 | 2-Ford | 0.30 | 0.01 |  |
| Recipient A | Fan A | High | 2.5 | 2-Ford | 0.32 | 0.02 |  |
| Recipient A | Fan A | High | 5 | 2-Ford | 0.31 | 0.00 |  |
| Recipient B | None | None | None | None | 1.00 | 0.15 |  |
| Recipient B | Fan A | Low | 2.5 | 2-Ford | 0.69 | 0.02 |  |
| Recipient B | Fan A | Low | 5 | 2-Ford | 0.62 | 0.03 |  |
| Recipient B | Fan A | High | 2.5 | 2-Ford | 0.61 | 0.04 |  |
| Recipient B | Fan A | High | 5 | 2-Ford | 0.51 | 0.03 |  |
| Recipient C | None | None | None | None | 1.00 | 0.06 |  |
| Recipient C | Fan A | Low | 2.5 | 2-Ford | 0.68 | 0.05 |  |
| Recipient C | Fan A | Low | 5 | 2-Ford | 0.43 | 0.11 |  |
| Recipient C | Fan A | High | 2.5 | 2-Ford | 0.55 | 0.07 |  |
| Recipient C | Fan A | High | 5 | 2-Ford | 0.34 | 0.03 |  |
|  |  |  |  |  |  |  |  |
| **Figure 5** | | | | | | | |
| Breather | Masking | Fan Model | Fan Speed | Filter Width (cm) | DIY Configuration | Normalized Mean (%) | Standard Deviation |
| Recipient A | Unmasked | No Unit | None | None | None | 1.00 | 0.40 |
| Recipient A | Unmasked | Fan A | Low | 2.5 | 2-Cube | 0.32 | 0.01 |
| Recipient A | Unmasked | Fan A | High | 2.5 | 2-Cube | 0.29 | 0.00 |
| Recipient A | Unmasked | Fan A | Low | 5 | 2-Cube | 0.26 | 0.01 |
| Recipient A | Unmasked | Fan A | High | 5 | 2-Cube | 0.17 | 0.01 |
| Recipient A | Masked | No Unit | None | None | None | 0.25 | 0.02 |
| Recipient A | Masked | Fan A | Low | 2.5 | 2-Cube | 0.11 | 0.01 |
| Recipient A | Masked | Fan A | High | 2.5 | 2-Cube | 0.09 | 0.00 |
| Recipient A | Masked | Fan A | Low | 5 | 2-Cube | 0.09 | 0.00 |
| Recipient A | Masked | Fan A | High | 5 | 2-Cube | 0.06 | 0.02 |
| Recipient B | Unmasked | No Unit | None | None | None | 1.00 | 0.15 |
| Recipient B | Unmasked | Fan A | Low | 2.5 | 2-Cube | 0.43 | 0.03 |
| Recipient B | Unmasked | Fan A | High | 2.5 | 2-Cube | 0.32 | 0.01 |
| Recipient B | Unmasked | Fan A | Low | 5 | 2-Cube | 0.32 | 0.01 |
| Recipient B | Unmasked | Fan A | High | 5 | 2-Cube | 0.24 | 0.01 |
| Recipient B | Masked | No Unit | None | None | None | 0.33 | 0.04 |
| Recipient B | Masked | Fan A | Low | 2.5 | 2-Cube | 0.16 | 0.01 |
| Recipient B | Masked | Fan A | High | 2.5 | 2-Cube | 0.10 | 0.01 |
| Recipient B | Masked | Fan A | Low | 5 | 2-Cube | 0.11 | 0.01 |
| Recipient B | Masked | Fan A | High | 5 | 2-Cube | 0.07 | 0.02 |
| Recipient C | Unmasked | No Unit | None | None | None | 1.00 | 0.06 |
| Recipient C | Unmasked | Fan A | Low | 2.5 | 2-Cube | 0.50 | 0.08 |
| Recipient C | Unmasked | Fan A | High | 2.5 | 2-Cube | 0.41 | 0.03 |
| Recipient C | Unmasked | Fan A | Low | 5 | 2-Cube | 0.33 | 0.03 |
| Recipient C | Unmasked | Fan A | High | 5 | 2-Cube | 0.25 | 0.01 |
| Recipient C | Masked | No Unit | None | None | None | 0.16 | 0.02 |
| Recipient C | Masked | Fan A | Low | 2.5 | 2-Cube | 0.09 | 0.01 |
| Recipient C | Masked | Fan A | High | 2.5 | 2-Cube | 0.05 | 0.00 |
| Recipient C | Masked | Fan A | Low | 5 | 2-Cube | 0.07 | 0.00 |
| Recipient C | Masked | Air King | High | 5 | 2-Cube | 0.05 | 0.01 |
|  |  |  |  |  |  |  |  |
| **Figure 6** | | | | | | | |
| Breather | Number & Type DIY | Fan Model | Filter Width (cm) | Location | Speed | Normalized Mean% | Standard Deviation |
| Recipient A | None | None | None | None | None | 1.00 | 0.40 |
| Recipient A | 1-Cube | Fan A | 2.5 | Front | Low | 0.38 | 0.03 |
| Recipient A | 1-Cube | Fan A | 2.5 | Front | High | 0.32 | 0.01 |
| Recipient A | 1-Cube | Fan A | 5 | Front | Low | 0.31 | 0.01 |
| Recipient A | 1-Cube | Fan A | 5 | Front | High | 0.26 | 0.02 |
| Recipient A | 1-Cube | Fan A | 2.5 | Back | Low | 0.67 | 0.23 |
| Recipient A | 1-Cube | Fan A | 2.5 | Back | High | 0.83 | 0.33 |
| Recipient A | 1-Cube | Fan A | 5 | Back | Low | 1.26 | 0.53 |
| Recipient A | 1-Cube | Fan A | 5 | Back | High | 0.27 | 0.02 |
| Recipient B | None | None | None | None | None | 1.00 | 0.15 |
| Recipient B | 1-Cube | Fan A | 2.5 | Front | Low | 0.47 | 0.03 |
| Recipient B | 1-Cube | Fan A | 2.5 | Front | High | 0.41 | 0.03 |
| Recipient B | 1-Cube | Fan A | 5 | Front | Low | 0.38 | 0.01 |
| Recipient B | 1-Cube | Fan A | 5 | Front | High | 0.32 | 0.01 |
| Recipient B | 1-Cube | Fan A | 2.5 | Back | Low | 0.72 | 0.03 |
| Recipient B | 1-Cube | Fan A | 2.5 | Back | High | 0.54 | 0.03 |
| Recipient B | 1-Cube | Fan A | 5 | Back | Low | 0.46 | 0.09 |
| Recipient B | 1-Cube | Fan A | 5 | Back | High | 0.40 | 0.01 |
| Recipient C | None | None | None | None | None | 1.00 | 0.06 |
| Recipient C | 1-Cube | Fan A | 2.5 | Front | Low | 1.06 | 0.07 |
| Recipient C | 1-Cube | Fan A | 2.5 | Front | High | 0.79 | 0.08 |
| Recipient C | 1-Cube | Fan A | 5 | Front | Low | 0.59 | 0.08 |
| Recipient C | 1-Cube | Fan A | 5 | Front | High | 0.52 | 0.02 |
| Recipient C | 1-Cube | Fan A | 2.5 | Back | Low | 0.47 | 0.06 |
| Recipient C | 1-Cube | Fan A | 2.5 | Back | High | 0.41 | 0.04 |
| Recipient C | 1-Cube | Fan A | 5 | Back | Low | 0.39 | 0.02 |
| Recipient C | 1-Cube | Fan A | 5 | Back | High | 0.35 | 0.11 |

**
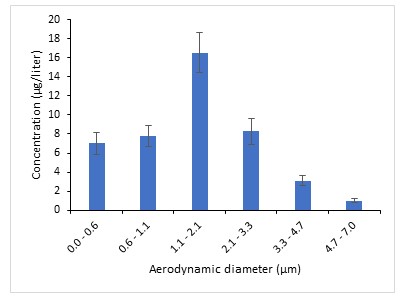
**

**Figure S1**: Size distribution of the exhaled aerosol. The aerosol had an average total mass concentration of approximately 45 µg/liter with a geometric mean diameter of 1.3 µm.

**
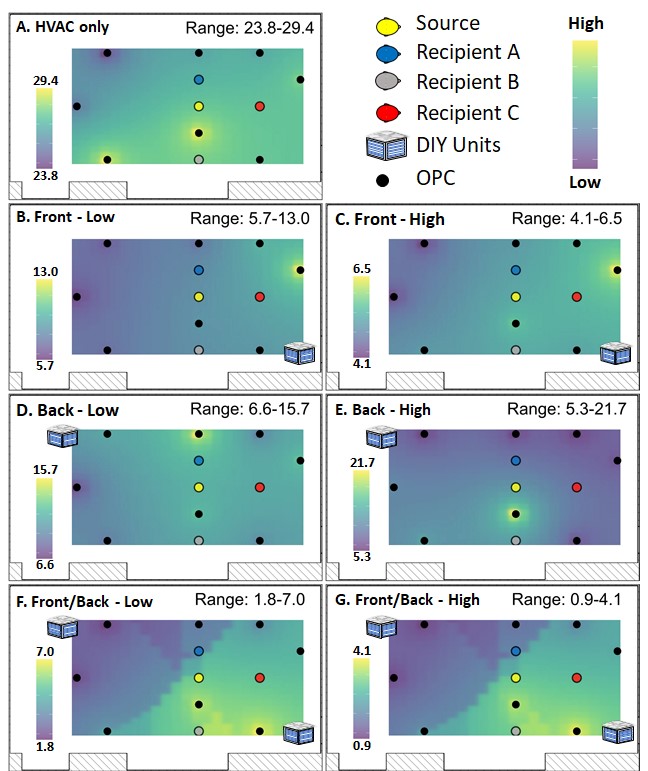
**

**Figure S2**: Spatial distribution of the mean aerosol mass concentration. The mean mass concentration measured by the OPC area samplers was quantified and overlain on the room diagrams. The particle concentration range (μg/m3) for each panel is listed in the top right corner. Each panel has a unique range and high and low concentration, with the highest concentration for that panel always represented by yellow and the lowest always represented by purple. The DIY air filtration cubes deployed were constructed with Fan A and 5 cm filters. (A) HVAC system set at 2 ACH without DIY air filtration cubes; (B) one DIY cube placed in the front of the room with the fan speed on low; (C) one DIY cube placed in the front with the fan speed on high; (D) one DIY cube placed in the back with the fan speed on low; (E) one DIY cube placed in the back with the fan speed on high; (F) two DIY cubes placed with one in front and one in back and with the fan speeds on low; and (G) two DIY cubes placed with one in front and one in back and with the fan speeds on high. The coloration has been normalized to the concentration range observed among all trials.
